# Supplementary material for: A potential transition from a concentrated to a generalized HIV epidemic: the case of Madagascar
Source: Infect Dis Poverty. 2023 Dec 7;12:112. doi: 10.1186/s40249-023-01164-2 (PMC10702038; doi:10.1186/s40249-023-01164-2)
Supplement: Supplementary file 1 — Additional file 1. Includes detailed supporting information about the dynamical ODE system corresponding to the model (Fig. 1), the R0 calculation, the strategy to conduct parameter estimation, model projections, and data sources: Additional Information for “A potential transition from a concentrated to a generalized HIV epidemic: the case of Madagascar”. [file 40249_2023_1164_MOESM1_ESM.pdf]

Additional File 1 for

A potential transition from a concentrated to a generalised HIV epidemic: the case of Madagascar

David Alonso and Xavier Vallès

David Alonso. E-mail: dalonso@ceab.csic.es

This PDF file includes detailed supplementary information about the dynamical ODE system corresponding to the model introduced in the main text (Fig 1), the R_0 calculation (SB46), the strategy to conduct parameter estimation, model projections, and data sources. It is also a guide to the code developed for this project. Both the C code and the data used are publicly available in (1)

Contents

A Model description **2**
A The **S**usceptible-**I**nfectious-**C**hronic-**A**IDS phase (**SICA**) Transmission Model . . . 2
B Stationary State . . . 3
B.1 The simple demographic model . . . 3
B.2 The expanded demographic model . . . 5
B.3 The full disease-transmission model . . . 5

B R0 through the next-generation matrix **5**

C Parameter estimation from demographic data **17**
A Fecundity and mortality rates . . . 17
A.1 Average mortality rates . . . 17
A.2 Recruitment Rates . . . 17
B The female population distribution . . . 21

D Model Validation **21**
A The likelihood function . . . 21
B The simple demographic model . . . 22
C The expanded demographic model . . . 23
D The full disease-transmission model . . . 25

E Projected Trajectories **25**

F Data Sources and Code **29**

A. Model description

A. The Susceptible-Infectious-Chronic-AIDS phase (SICA) Transmission Model. We represent the temporal dynamics of disease spread by a set of ordinary differential equations, where, as in any other sexually transmitted infection, disease progression (from the infected to the acute or AIDS phase) depends on sexual contacts between males and females (2). The female-male coupled system is organized into the male and the female submodels. These separate submodels are coupled through the force of infection or transmission rate, that is, the per capita rate at which male (or female) susceptible individuals acquire the infection through sexual contacts from female (or male) infectious individuals. This transmission rate takes into account the number of sexual contacts per year of an average male (or female) individuals, β_Y (or β_X), the distinct probability of transmission from infectious females to males (or from infectious males to females) (3), p_{YX} (or p_{XY}), and the effective population fractions of infectious females (or males), x (or y) (4). Notice also that basal female contact rate, β_X, is corrected by a factor $\eta > 1$ for sexual workers to indicate the higher contact rates characterizing this activity.

The male subsystem reads:

$$\begin{aligned}
\frac{dY_S}{dt} &= F_Y - \beta_Y\, p_{YX}\, x\, Y_S - \delta\, Y_S \\
\frac{dY_I}{dt} &= \beta_Y\, p_{YX}\, x\, Y_S - \delta\, Y_I - \gamma\, Y_I \\
\frac{dY_C}{dt} &= \gamma\, Y_I - \mu\, Y_C - \delta\, Y_C \\
\frac{dY_A}{dt} &= \mu\, Y_C - (1+m)\, \delta\, Y_A
\end{aligned} \qquad \text{[SA1]}$$

where the total effective fraction of infectious females is a weighted average of the effective infectious fractions including young, x_0, and adult women, x_1:

$$x = f_0\, x_0 + (1 - f_0)\, x_1 \qquad \text{[SA2]}$$

where f_0 is the fraction of total sexual encounters a male has with young females. Effective infectious fractions of females should account for differential transmission of the disease from females either in the highly infectious group or the chronic group:

$$x_0 = \frac{f_W\, (\chi\, W_I^{(0)} + W_C^{(0)}) + (1 - f_W)(\chi\, X_I^{(0)} + X_C^{(0)})}{N_f} \qquad \text{[SA3]}$$

$$x_1 = \frac{f_W\, (\chi\, W_I^{(1)} + W_C^{(1)}) + (1 - f_W)(\chi\, X_I^{(1)} + X_C^{(1)})}{N_f} \qquad \text{[SA4]}$$

where f_W if the fraction of male sexual contacts with women who are sexual workers, and N_f is the total female population.

The female subsystem accounts for the progression of the disease in the four female groups, which all follow the same four-equation scheme. Equations for young females that are not sex workers are given by:

$$\begin{aligned}
\frac{dX_S^{(0)}}{dt} &= F_X - \beta_X\, p_{XY}\, y\, X_S^{(0)} - \delta\, X_S^{(0)} - \alpha\, X_S^{(0)} - \sigma^0\, X_S^{(0)} + \sigma_r^0\, W_S^{(0)} \\
\frac{dX_I^{(0)}}{dt} &= \beta_X\, p_{XY}\, y\, X_S^{(0)} - \delta\, X_I^{(0)} - \alpha\, X_I^{(0)} - \gamma\, X_I^{(0)} - \sigma^0\, X_I^{(0)} + \sigma_r^0\, W_I^{(0)} \\
\frac{dX_C^{(0)}}{dt} &= \gamma\, X_I^{(0)} - \mu\, X_C^{(0)} - \delta\, X_C^{(0)} - \alpha\, X_C^{(0)} - \sigma^0\, X_C^{(0)} + \sigma_r^0\, W_C^{(0)} \\
\frac{dX_A^{(0)}}{dt} &= \mu\, X_C^{(0)} - (1+m)\, \delta\, X_A^{(0)} - \alpha\, X_A^{(0)} - \sigma^0\, X_A^{(0)} + \sigma_r^0\, W_A^{(0)}
\end{aligned} \qquad \text{[SA5]}$$

The four basic equations for old/adult non-sex-working females are:

$$\begin{aligned}
\frac{dX_S^{(1)}}{dt} &= -\beta_X\, p_{XY}\, y\, X_S^{(1)} - \delta\, X_S^{(1)} + \alpha\, X_S^{(0)} - \sigma^1\, X_S^{(1)} + \sigma_r^1\, W_S^{(1)} \\
\frac{dX_I^{(1)}}{dt} &= \beta_X\, p_{XY}\, y\, X_S^{(1)} - \delta\, X_I^{(1)} + \alpha\, X_I^{(0)} - \gamma\, X_I^{(0)} - \sigma^1\, X_I^{(1)} + \sigma_r^1\, W_I^{(1)} \\
\frac{dX_C^{(1)}}{dt} &= \gamma\, X_I^{(1)} - \mu\, X_C^{(1)} - \delta\, X_C^{(1)} + \alpha\, X_C^{(0)} - \sigma^1\, X_C^{(1)} + \sigma_r^1\, W_C^{(1)} \\
\frac{dX_A^{(1)}}{dt} &= \mu\, X_C^{(1)} - (1+m)\, \delta\, X_A^{(1)} + \alpha\, X_A^{(0)} - \sigma^1\, X_A^{(1)} + \sigma_r^1\, W_A^{(1)}
\end{aligned} \qquad \text{[SA6]}$$

Likewise, the four equations for young, female sex workers read:

$$\begin{aligned}
\frac{dW_S^{(0)}}{dt} &= -\beta_X\, p_{XY}\,(1+\eta)\,y\,W_S^{(0)} - \delta\,W_S^{(0)} - \alpha\,W_S^{(0)} - \sigma_r^0\,W_S^{(0)} + \sigma^0\,X_S^{(0)} \\
\frac{dW_I^{(0)}}{dt} &= \beta_X\, p_{XY}\,(1+\eta)\,y\,W_S^{(0)} - \delta\,W_I^{(0)} - \alpha\,W_I^{(0)} - \gamma\,W_I^{(0)} - \sigma_r^0\,W_I^{(0)} + \sigma^0\,X_I^{(0)} \\
\frac{dW_C^{(0)}}{dt} &= \gamma\,W_I^{(0)} - \mu\,W_C^{(0)} - \delta\,W_C^{(0)} - \alpha\,W_C^{(0)} - \sigma_r^0\,W_C^{(0)} + \sigma^0\,X_C^{(0)} \\
\frac{dW_A^{(0)}}{dt} &= \mu\,W_C^{(0)} - (1+m)\,\delta\,W_A^{(0)} - \alpha\,W_A^{(0)} - \sigma_r^0\,W_A^{(0)} + \sigma^0\,X_A^{(0)} \qquad \text{[SA7]}
\end{aligned}$$

Finally, the equations for old/adult female sex workers are represented by:

$$\begin{aligned}
\frac{dW_S^{(1)}}{dt} &= -\beta_X\, p_{XY}\,(1+\eta)\,y\,W_S^{(1)} - \delta\,W_S^{(1)} + \alpha\,W_S^{(0)} - \sigma_r^1\,W_S^{(1)} + \sigma^1\,X_S^{(1)} \\
\frac{dW_I^{(1)}}{dt} &= \beta_X\, p_{XY}\,(1+\eta)\,y\,W_S^{(1)} - \delta\,W_I^{(1)} + \alpha\,W_I^{(0)} - \gamma\,W_I^{(1)} - \sigma_r^1\,W_I^{(1)} + \sigma^1\,X_I^{(1)} \\
\frac{dW_C^{(1)}}{dt} &= \gamma\,W_I^{(1)} - \mu\,W_C^{(1)} - \delta\,W_C^{(1)} + \alpha\,W_C^{(0)} - \sigma_r^1\,W_C^{(1)} + \sigma^1\,X_C^{(1)} \\
\frac{dW_A^{(1)}}{dt} &= \mu\,W_C^{(1)} - (1+m)\,\delta\,W_A^{(1)} + \alpha\,W_A^{(0)} - \sigma_r^1\,W_A^{(1)} + \sigma^1\,X_A^{(1)} \qquad \text{[SA8]}
\end{aligned}$$

As you see in Eqs (SA5)-(SA8), the force of infection of females includes a factor, the total effective fraction of infectious males, y, which is given by:

$$y = \frac{\chi\,Y_I + Y_C}{N_m} \qquad \text{[SA9]}$$

where N_m is the total male population. Total populations, N_m and N_f, also change dynamically. They are written in terms of sums over the different disease stages and groups:

$$\begin{aligned}
N_m &= Y_S + Y_I + Y_C + Y_A \\
N_f &= \hat{X}_S + \hat{X}_I + \hat{X}_C + \hat{X}_A \qquad \text{[SA10]}
\end{aligned}$$

where

$$\begin{aligned}
\hat{X}_a &= X_a^{(0)} + X_a^{(1)} + W_a^{(0)} + W_a^{(1)} \\
a &\in \{S, I, C, A\} \qquad \text{[SA11]}
\end{aligned}$$

Total effective infectious fractions, x an y, are the link between female and male disease dynamics. These dynamically changing variables are not true fractions as they are not constrained between 0 and 1 because they include the factor $\chi > 1$, which measures the increase in transmission through sexual encounters with infectious individuals in the acute phase compared to the chronic phase (they would be only proper fractions if χ were 1). In any case, these normalized variables, x (or y), times p_{XY} (or p_{YX}) determine the probability rate of acquiring the infection per sexual encounter.

The full list of model paramaters for the SICA model is given in Table SA1 along the references used to establish plausible parameter ranges.

B. Stationary State.

B.1. The simple demographic model. The SICA model extremely simplifies population demography. In the absence of disease transmission, one can check that female and male subpopulations evolve in time according to the following system:

$$\begin{aligned}
\frac{dY}{dt} &= F_Y - \delta_Y Y \\
\frac{dX^{(0)}}{dt} &= F_X - \sigma^0 X^{(0)} + \sigma_r^0 W^{(0)} - \alpha X^{(0)} - \delta_X X^{(0)} \\
\frac{dW^{(0)}}{dt} &= \sigma^0 X^{(0)} - \sigma_r^0 W^{(0)} - \alpha W^{(0)} - \delta_X W^{(0)} \\
\frac{dX^{(1)}}{dt} &= \alpha X^{(0)} + \sigma_r^0 W^{(1)} - \sigma^0 X^{(1)} - \delta_X X^{(1)} \\
\frac{dW^{(1)}}{dt} &= \alpha W^{(0)} + \sigma^0 X^{(1)} - \sigma_r^0 W^{(1)} - \delta_X W^{(1)} \qquad \text{[SA12]}
\end{aligned}$$

Table SA1. Model Parameters for the $SICA$ model. V_0 and V_1 define reasonable parameter ranges for each parameter value. Subscripts X and Y stand for female and male, respectively. YF, young female; AF, adult female; SW, sexual worker. All rates are given in year^{-1}. Mortality and recruitment rates are directly estimated from life table information. Transmission probabilities are given per coital act and represent basal values. The χ factor controls how much these are incremented when a healthy individual has a sexual encounter with an individual in the acute infection phase (I). The inverse of transition rates between stages (for instance, $1/\gamma$ and $1/\mu$) can be regarded as an average residence time in that stage once you are in it. Consequently, the range for the average length of the infection phase (I) (see γ values) is considered to be between 2 months and 2 years (an average of 70 days after (5)). Likewise, the average length of the C phase (see μ values) may span from 5 to 20 years (an average of 9.8 years after (6)), and the average duration of a young age for females (see α values) is considered to be between 8 and 12 years after entering full active sexual life (at the age of 15).

**Model Parameter**	**Symbol**	V_0	V_1	**Refs.**
Demographic parameters				
Recruitment rate into active sexual age	F_Y	0.00	5 10^4	(7)
Recruitment rate into active sexual age	F_X	0.00	5 10^4	(7)
Natural mortality percapita rate (X)	δ_X	0.01	0.05	(7)
Natural mortality percapita rate (Y)	δ_Y	0.01	0.05	(7)
Aging rate into the AF stage	α	0.08	0.12	
Transition rate into the SW stage (YF)	σ^0	0.00	0.05	
Reverse rate from the SW stage (YF)	σ_r^0	0.00	0.05	
Transition rate into the SW stage (AF)	σ^1	0.00	0.05	
Reverse rate from the SW stage (AF)	σ_r^1	0.00	0.05	
Disease transmission parameters				
Total male sexual contact rate	β_Y	96.0	120.0	(8)
Total female sexual contact rate	β_X	96.0	120.0	(8)
Sexual worker β_X factor [$(1+\eta)\beta_X$]	η	0.00	19.00	
Male-to-Female transmission probability	p_{XY}	0.00	0.005	(8–10)
Female-to-Male transmission probability	p_{YX}	0.00	0.001	(8–10)
Transition rate from I into C stage	γ	0.5	6.00	(5)
Transition rate from C into A stage	μ	0.05	0.20	(6)
Disease-induced mortality factor [$(1+m)\delta$]	m	0.00	99.00	
Infectious phase (I) transmission factor	χ	1.00	100.00	(11)
Male sexual preference factor (for SW)	f_W	0.00	0.99	
Male sexual preference factor (for YF)	f_0	0.00	0.99	

Further simplification is reached if we sum up over female equations. In that case, female and male adult populations are just tracked by the simple system:

$$\frac{dY}{dt} = F_Y - \delta_Y Y$$
$$\frac{dX}{dt} = F_X - \delta_X X \qquad \text{[SA13]}$$

The stationary state, which is also the disease-free equilibrium, is given by:

$$Y^\star = \frac{F_Y}{\delta_Y}$$
$$X^\star = \frac{F_X}{\delta_X} \qquad \text{[SA14]}$$

B.2. The expanded demographic model. The system given by Eqs. (SA12) involves decoupled dynamics of females and males. The first Eq. in (SA12) has been already solved for equilibrium. The last four Eqs in (SA12) describe adult female dynamics and are linear ODEs. Therefore, the following stationary equilibrium can be easily calculated. We write here the proportion of females in each of the groups. We use lower-case letters to note fractions or proportions with respect to total female population at equilibrium (F_X/δ_X). First, the young female proportions at equilibrium are:

$$x^{(0)\star} = \frac{(\sigma_r^0 + \delta_X + \alpha)\,\delta_X}{(\delta_X + \alpha)\,(\sigma^0 + \sigma_r^0 + \delta_X + \alpha)}$$
$$w^{(0)\star} = \frac{\sigma_0\,\delta_X}{(\delta_X + \alpha)\,(\sigma^0 + \sigma_r^0 + \delta_X + \alpha)} \qquad \text{[SA15]}$$

By looking at the equations for the temporal evolution of older females, we see that their equilibrium fractions can be easily written in terms of $x^{(0)\star}$ and $w^{(0)\star}$:

$$w^{(1)\star} = \frac{\sigma^1}{\sigma_r^1 + \sigma_1 + \delta_X} + \frac{\sigma_0 - \sigma^1}{\sigma_r^1 + \sigma_1 + \delta_X}\,x^{(0)\star} + \frac{\sigma_r^0 + \sigma_1 + \delta_X}{\sigma_r^1 + \sigma_1 + \delta_X}\,w^{(0)\star}$$
$$x^{(1)\star} = 1 - w^{(1)\star} - x^{(0)\star} - w^{(0)\star} \qquad \text{[SA16]}$$

B.3. The full disease-transmission model. The full system given by Eqs (SA1)-(SA8) is not linear in the dynamical variables. However, given a constant infectious effective fraction for females x , male equations are very simple to solve (see Eqs. (SA1):

$$Y_S^\star = \frac{F_Y}{\beta_Y p_0 x + \delta_Y}$$
$$Y_I^\star = \frac{\beta_Y p_0 x}{\beta_Y p_0 x + \delta_Y}\,\frac{F_Y}{\delta_X + \gamma}$$
$$Y_L^\star = \frac{\gamma}{\mu + \delta_Y}\,\frac{\beta_Y p_0 x}{\beta_Y p_0 x + \delta_Y}\,\frac{F_Y}{\delta_X + \gamma}$$
$$Y_D^\star = \frac{\mu}{\mu + \delta_Y}\,\frac{\gamma}{(1+m)\delta_Y}\,\frac{\beta_Y p_0 x}{\beta_Y p_0 x + \delta_Y}\,\frac{F_Y}{\delta_X + \gamma} \qquad \text{[SA17]}$$

Notice that the stationary state calculated above depends on x. Likewise, given a constant infectious effective fraction for males y (see Eqs. (SA5)-(SA8)), the 16 different female population types have an equilibrium that depends on y. This steady state can be readily calculated by equating Eqs. (SA5)-(SA8) to zero, which gives rise to a linear system of 16 equations and 16 unknowns (see code in the paper github site for a full specification of this solution). We can thus calculate both the expected effective fraction of infectious males y for a given constant effective fraction of infectious females x, and the expected effective fraction of infectious females x for a given constant effective fraction of infectious males y. The intersection of these two curves, x and y, defines the stationary effective fractions $(x^\star, y^\star)$ (Fig SA1), which then can be used to identify the complete stationary state through Eqs. (SA17) and the analogous equations for females (12). As expected, see Fig SA2, the temporal evolution of total prevalence tends to an asymptotic stationary state (horizontal broken orange line). Under the assumption of constant recruitment of new susceptible individuals into the adult populations, sex-age structured compartmental mathematical models for sexual-transmitted infections with constant disease-induced mortality can be shown to be well-poised and feasible (13).

B. R0 through the next-generation matrix

Diekmann *et al.* (14) introduced a general method to calculate the basic reproduction number R_0 for compartmental models of disease transmission, i.e. epidemic models defined as a system of ordinary differential equations (ODEs). The method starts by identifying the so-called infection subsystem, i.e. the system of equations affecting only infected individuals. The linearization of this subsystem around the disease-free state gives us information about the growth rate of the infected population when the disease starts with a few infected people in an otherwise healthy population. These authors showed that R_0 is the leading

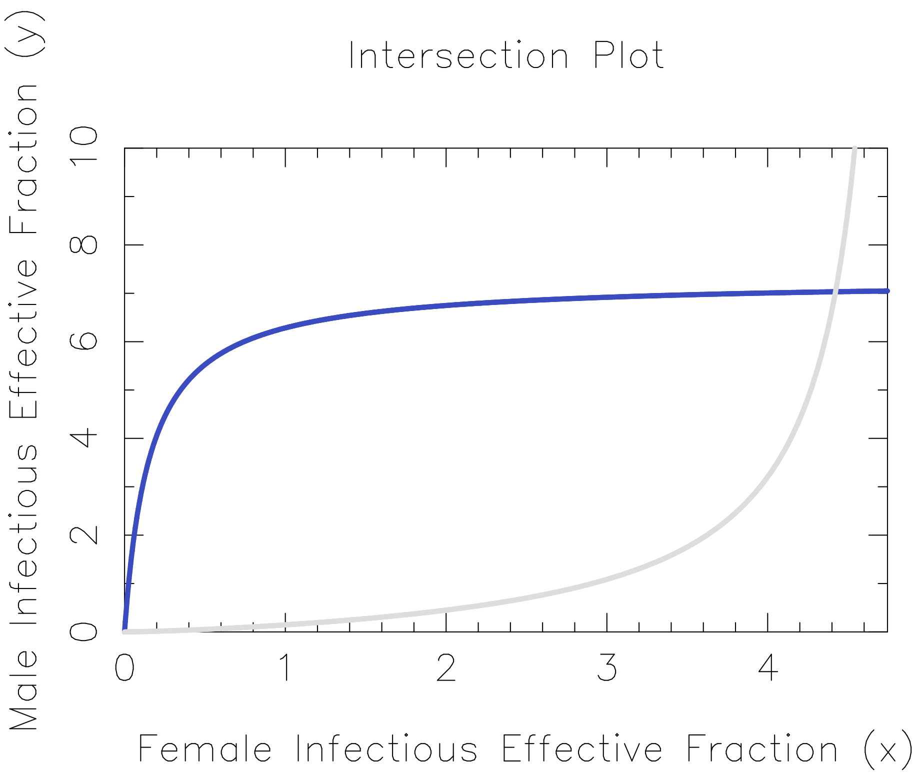

Fig. SA1. The intersection of these two curves, $y = F(x)$ and $y = G^{-1}(x)$, defines the stationary effective fractions.

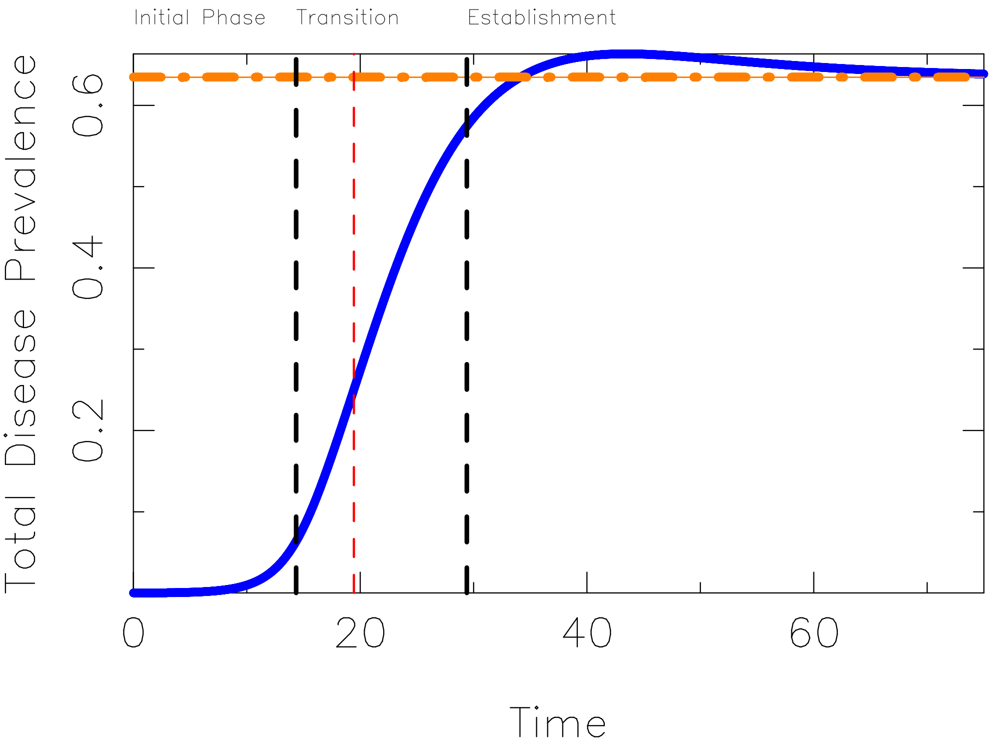

Fig. SA2. Temporal evolution of model HIV total prevalence when using constant parameters. The two vertical broken black lines define the three different phases as explained in the main text. The vertical broken red line defines the turning point. Since parameters are kept constant, the system reaches an asymptotic stationary state (horizontal broken orange line). The initial condition corresponds to a population with 10 infected sexual workers in a total population of sexual workers of 1000 within a total adult population (men and women) of 100000.

eigenvalue of the so-called “next generation matrix" (NGM), which can be built from the linearized infection subsystem. Here we outline the derivation of NGM and the calculation of R_0 as the leading eigenvalue of this matrix.

Using the main ODE system Eqs. (SA1)-(SA8), we can extract the equations affecting only infected individuals. We obtain two equation for males:

$$\begin{aligned} \frac{dY_I}{dt} &= \beta_Y \, p_{YX} \, x \, Y_S - (\gamma + \delta_Y) \, Y_I \\ \frac{dY_L}{dt} &= \gamma \, Y_I - (\mu + \delta_Y) \, Y_L \end{aligned} \quad \text{[SB1]}$$

where x is the female infectious effective fraction (see Eq (SA2)). We also obtain 8 equations for the different types of female infected individuals:

$$\begin{aligned} \frac{dX_I^{(0)}}{dt} &= \beta_X \, p_{XY} \, y \, X_S^{(0)} - (\alpha + \gamma + \sigma^0 + \delta_X) \, X_I^{(0)} + \sigma_r^0 \, W_I^{(0)} \\ \frac{dX_L^{(0)}}{dt} &= \gamma \, X_I^{(0)} - (\mu + \alpha + \sigma^0 + \delta_X) \, X_L^{(0)} + \sigma_r^0 \, W_L^{(0)} \end{aligned} \quad \text{[SB2]}$$

$$\begin{aligned} \frac{dW_I^{(0)}}{dt} &= \beta_X \, (1 - \eta) \, p_{XY} \, y \, W_S^{(0)} - (\alpha + \gamma + \sigma_r^0 + \delta_X) \, W_I^{(0)} + \sigma^0 \, X_I^{(0)} \\ \frac{dW_L^{(0)}}{dt} &= \gamma \, W_I^{(0)} - (\mu + \alpha + \sigma_r^0 + \delta_X) \, W_L^{(0)} + \sigma^0 \, X_L^{(0)} \end{aligned} \quad \text{[SB3]}$$

$$\begin{aligned} \frac{dX_I^{(1)}}{dt} &= \beta_X \, p_{XY} \, y \, X_S^{(1)} + \alpha \, X_I^{(0)} - (\gamma + \sigma^1 + \delta_X) \, X_I^{(1)} + \sigma_r^1 \, W_I^{(1)} \\ \frac{dX_L^{(1)}}{dt} &= \gamma \, X_I^{(1)} + \alpha \, X_L^{(0)} - (\mu + \sigma^1 + \delta_X) \, X_L^{(1)} + \sigma_r^1 \, W_L^{(1)} \end{aligned} \quad \text{[SB4]}$$

$$\begin{aligned} \frac{dW_I^{(1)}}{dt} &= \beta_X \, (1 - \eta) \, p_{XY} \, y \, W_S^{(1)} + \alpha \, W_I^{(0)} - (\gamma + \sigma_r^0 + \delta_X) \, W_I^{(1)} + \sigma^1 \, X_I^{(1)} \\ \frac{dW_L^{(1)}}{dt} &= \gamma \, W_I^{(1)} + \alpha \, W_L^{(0)} - (\mu + \alpha + \sigma_r^1 + \delta_X) \, W_L^{(1)} + \sigma^1 \, X_L^{(1)} \end{aligned} \quad \text{[SB5]}$$

where y is the male infectious effective fraction (see Eq (SA9)).

We notice now that, in the absence of disease, the full system collapses into the simple demographic model described by (Eqs SA12). The disease-free equilibrium therefore can be simply characterized by:

$$N_X = \frac{F_X}{\delta_X} \qquad N_Y^\star = \frac{F_Y}{\delta_Y} \quad \text{[SB6]}$$

The linearization of the infection subsystem around this disease-free state can be represented with the help of two matrices, the transmission matrix $\mathbf{T}$, and the transition matrix $\mathbf{\Sigma}$. First, we define the state vector $\vec{x}$ as

$$\vec{x} = \left(Y_I, Y_L, X_I^{(0)}, W_I^{(0)}, X_I^{(1)}, W_I^{(1)}, X_L^{(0)}, W_L^{(0)}, X_L^{(1)}, W_L^{(1)} \right) \quad \text{[SB7]}$$

One can check that the system given by Eqs (SB1)-(SB5), around the disease-free state, can be readily written as:

$$\frac{d\vec{x}}{dt} = (\mathbf{T} + \mathbf{\Sigma}) \; \vec{x} \quad \text{[SB8]}$$

where the matrices $\mathbf{T}$ and $\mathbf{\Sigma}$ are defined in a proper way. If further notation for compound parameters is adopted:

$$\begin{aligned} \Pi_Y^\star &= \frac{N_Y^\star}{N_X^\star} = \frac{F_Y}{F_X} \frac{\delta_X}{\delta_Y} \\ \Pi_X^\star &= \frac{N_X^\star}{N_Y^\star} = \frac{F_X}{F_Y} \frac{\delta_Y}{\delta_X} \end{aligned} \quad \text{[SB9]}$$

$$\begin{aligned} \beta_{YX} &= \beta_Y \, p_{YX} \, \Pi_Y^\star \\ \beta_{XY} &= \beta_X \, p_{XY} \, \Pi_X^\star \\ \beta_{XY}^{(W)} &= \beta_X \, (1 + \eta) \, p_{XY} \, \Pi_X^\star \end{aligned} \quad \text{[SB10]}$$

$$
\begin{aligned}
f_1 &= 1 - f_0 \\
f_X &= 1 - f_W
\end{aligned}
\qquad \text{[SB11]}
$$

and the average residence times in each of the infectious stages:

$$T_{Y_I} = \frac{1}{\gamma + \delta_Y} \qquad \text{[SB12]}$$

$$T_{Y_L} = \frac{1}{\mu + \delta_Y} \qquad \text{[SB13]}$$

$$T_{X_I^{(0)}} = \frac{1}{\gamma + \alpha + \sigma^0 + \delta_X} \qquad \text{[SB14]}$$

$$T_{X_I^{(1)}} = \frac{1}{\gamma + \sigma^1 + \delta_X} \qquad \text{[SB15]}$$

$$T_{W_I^{(0)}} = \frac{1}{\gamma + \alpha + \sigma_r^0 + \delta_X} \qquad \text{[SB16]}$$

$$T_{W_I^{(1)}} = \frac{1}{\gamma + \sigma_r^1 + \delta_X} \qquad \text{[SB17]}$$

$$T_{X_L^{(0)}} = \frac{1}{\mu + \alpha + \sigma^0 + \delta_X} \qquad \text{[SB18]}$$

$$T_{X_L^{(1)}} = \frac{1}{\mu + \sigma^1 + \delta_X} \qquad \text{[SB19]}$$

$$T_{W_L^{(0)}} = \frac{1}{\mu + \alpha + \sigma_r^0 + \delta_X} \qquad \text{[SB20]}$$

$$T_{W_L^{(1)}} = \frac{1}{\mu + \sigma_r^1 + \delta_X}\,, \qquad \text{[SB21]}$$

then the matrices $\mathbf{T}$ and $\mathbf{\Sigma}$ are written as follows:

$$
\mathbf{T} = \begin{bmatrix}
0 & 0 & \beta_{YX} f_0 f_X \chi & \beta_{YX} f_0 f_W \chi & \beta_{YX} f_1 f_X \chi & \beta_{YX} f_1 f_W \chi & \beta_{YX} f_0 f_X & \beta_{YX} f_0 f_W & \beta_{YX} f_1 f_X & \beta_{YX} f_1 f_W \\
0 & 0 & 0 & 0 & 0 & 0 & 0 & 0 & 0 & 0 \\
\beta_{XY}\chi & \beta_{XY} & 0 & 0 & 0 & 0 & 0 & 0 & 0 & 0 \\
\beta_{XY}^{(W)}\chi & \beta_{XY}^{(W)} & 0 & 0 & 0 & 0 & 0 & 0 & 0 & 0 \\
\beta_{XY}\chi & \beta_{XY} & 0 & 0 & 0 & 0 & 0 & 0 & 0 & 0 \\
\beta_{XY}^{(W)}\chi & \beta_{XY}^{(W)} & 0 & 0 & 0 & 0 & 0 & 0 & 0 & 0 \\
0 & 0 & 0 & 0 & 0 & 0 & 0 & 0 & 0 & 0 \\
0 & 0 & 0 & 0 & 0 & 0 & 0 & 0 & 0 & 0 \\
0 & 0 & 0 & 0 & 0 & 0 & 0 & 0 & 0 & 0 \\
0 & 0 & 0 & 0 & 0 & 0 & 0 & 0 & 0 & 0
\end{bmatrix}
\qquad \text{[SB22]}
$$

$$
\mathbf{\Sigma} = \begin{bmatrix}
-\frac{1}{T_{Y_I}} & 0 & 0 & 0 & 0 & 0 & 0 & 0 & 0 & 0 \\
\gamma & -\frac{1}{T_{Y_L}} & 0 & 0 & 0 & 0 & 0 & 0 & 0 & 0 \\
0 & 0 & -\frac{1}{T_{X_I^{(0)}}} & \sigma_0^r & 0 & 0 & 0 & 0 & 0 & 0 \\
0 & 0 & \sigma_0 & -\frac{1}{T_{W_I^{(0)}}} & 0 & 0 & 0 & 0 & 0 & 0 \\
0 & 0 & \alpha & 0 & -\frac{1}{T_{X_I^{(1)}}} & \sigma_r^1 & 0 & 0 & 0 & 0 \\
0 & 0 & 0 & \alpha & \sigma^1 & -\frac{1}{T_{W_I^{(1)}}} & 0 & 0 & 0 & 0 \\
0 & 0 & \gamma & 0 & 0 & 0 & -\frac{1}{T_{X_L^{(0)}}} & \sigma_r^0 & 0 & 0 \\
0 & 0 & 0 & \gamma & 0 & 0 & \sigma^0 & -\frac{1}{T_{W_L^{(0)}}} & 0 & 0 \\
0 & 0 & 0 & 0 & \gamma & 0 & \alpha & 0 & -\frac{1}{T_{X_L^{(1)}}} & \sigma_r^1 \\
0 & 0 & 0 & 0 & 0 & \gamma & 0 & \alpha & \sigma^1 & -\frac{1}{T_{W_L^{(1)}}}
\end{bmatrix}
\qquad \text{[SB23]}
$$

It is shown that R_0 is the dominant eigenvalue of the NGM, which is precisely defined in terms of the previous $\mathbf{T}$ and $\mathbf{\Sigma}$ matrices as follows:

$$\mathbf{K} = -\mathbf{E}^T\,\mathbf{T}\,\mathbf{\Sigma}^{-1}\,\mathbf{E} \tag{SB24}$$

where $\mathbf{E}$ is an auxiliary matrix built from the transmission matrix $\mathbf{T}$, consisting of unit column vectors e_i for all i such that ith row of $\mathbf{T}$ is not identically zero:

$$\mathbf{E} = \begin{bmatrix} 1 & 0 & 0 & 0 & 0 \\ 0 & 0 & 0 & 0 & 0 \\ 0 & 1 & 0 & 0 & 0 \\ 0 & 0 & 1 & 0 & 0 \\ 0 & 0 & 0 & 1 & 0 \\ 0 & 0 & 0 & 0 & 1 \\ 0 & 0 & 0 & 0 & 0 \\ 0 & 0 & 0 & 0 & 0 \\ 0 & 0 & 0 & 0 & 0 \\ 0 & 0 & 0 & 0 & 0 \end{bmatrix} \tag{SB25}$$

Interestingly, in our case it is not necessary to explicitly calculate the inverse of the $\mathbf{\Sigma}$ matrix because the elements $\left[-\left(\mathbf{\Sigma}^{-1}\right)\right]_{ij}$ have a clear epidemiological interpretation: the expected time that individuals currently in state j will spend in state i before dying or transitioning into a non-infectious stage. This interpretation calculate allows a straightforward calculation of the whole $-\mathbf{\Sigma}^{-1}$ matrix which results into Eq. (SB27).

We now calculate the matrix product, $\mathbf{K_L} = \mathbf{T}(-\mathbf{\Sigma}^{-1})$:

$$\mathbf{K_L} = \begin{bmatrix} 0 & 0 & k_{(1,3)} & k_{(1,4)} & k_{(1,5)} & k_{(1,6)} & k_{(1,7)} & k_{(1,8)} & k_{(1,9)} & k_{(1,10)} \\ 0 & 0 & 0 & 0 & 0 & 0 & 0 & 0 & 0 & 0 \\ k_{(3,1)} & k_{(3,2)} & 0 & 0 & 0 & 0 & 0 & 0 & 0 & 0 \\ k_{(4,1)} & k_{(4,2)} & 0 & 0 & 0 & 0 & 0 & 0 & 0 & 0 \\ k_{(5,1)} & k_{(5,2)} & 0 & 0 & 0 & 0 & 0 & 0 & 0 & 0 \\ k_{(6,1)} & k_{(6,2)} & 0 & 0 & 0 & 0 & 0 & 0 & 0 & 0 \\ 0 & 0 & 0 & 0 & 0 & 0 & 0 & 0 & 0 & 0 \\ 0 & 0 & 0 & 0 & 0 & 0 & 0 & 0 & 0 & 0 \\ 0 & 0 & 0 & 0 & 0 & 0 & 0 & 0 & 0 & 0 \\ 0 & 0 & 0 & 0 & 0 & 0 & 0 & 0 & 0 & 0 \end{bmatrix} \tag{SB26}$$

where the 18 non-null elements k_{ij} are patiently and carefully defined in order—see Eqs (SB28)-(SB43):

$$-\boldsymbol{\Sigma}^{-1} = \begin{bmatrix} T_{Y_I} & 0 & 0 & 0 & 0 & 0 & 0 & 0 & 0 & 0 \\ \gamma T_{Y_I} T_{Y_L} & T_{Y_L} & 0 & 0 & 0 & 0 & 0 & 0 & 0 & 0 \\ 0 & 0 & T_{X_I^{(0)}} & \sigma_0^r T_{W_I^{(0)}} T_{X_I^{(0)}} & 0 & 0 & 0 & 0 & 0 & 0 \\ 0 & 0 & \sigma_0 T_{X_I^{(0)}} T_{W_I^{(0)}} & T_{W_I^{(0)}} & 0 & 0 & 0 & 0 & 0 & 0 \\ 0 & 0 & \alpha T_{X_I^{(0)}} T_{X_I^{(1)}} & 0 & T_{X_I^{(1)}} & \sigma_r^1 T_{W_I^{(1)}} T_{X_I^{(1)}} & 0 & 0 & 0 & 0 \\ 0 & 0 & 0 & \alpha T_{W_I^{(0)}} T_{W_I^{(1)}} & \sigma^1 T_{X_I^{(1)}} T_{W_I^{(1)}} & T_{W_I^{(1)}} & 0 & 0 & 0 & 0 \\ 0 & 0 & \gamma T_{X_I^{(0)}} T_{X_L^{(0)}} & 0 & 0 & 0 & T_{X_L^{(0)}} & \sigma_r^0 T_{W_L^{(0)}} T_{X_L^{(0)}} & 0 & 0 \\ 0 & 0 & 0 & \gamma T_{W_I^{(0)}} T_{W_L^{(0)}} & 0 & 0 & \sigma^0 T_{X_L^{(0)}} T_{W_L^{(0)}} & T_{W_L^{(0)}} & 0 & 0 \\ 0 & 0 & 0 & 0 & \gamma T_{X_I^{(1)}} T_{X_L^{(1)}} & 0 & \alpha T_{X_L^{(0)}} T_{X_L^{(1)}} & 0 & T_{X_L^{(1)}} & \sigma_r^1 T_{W_L^{(1)}} T_{X_L^{(1)}} \\ 0 & 0 & 0 & 0 & 0 & \gamma T_{X_I^{(1)}} T_{W_L^{(1)}} & 0 & \alpha T_{W_L^{(0)}} T_{W_L^{(1)}} & \sigma^1 T_{X_L^{(1)}} T_{W_L^{(1)}} & T_{W_L^{(1)}} \end{bmatrix} \quad \text{[SB27]}$$

$$k_{(3,1)} = \beta_{XY}\chi T_{Y_I} + \beta_{XY}\gamma T_{Y_I} T_{Y_L} \tag{SB28}$$

$$k_{(3,2)} = \beta_{XY} T_{Y_L} \tag{SB29}$$

$$k_{(4,1)} = \beta_{XY}^{(W)}\chi T_{Y_I} + \beta_{XY}^{(W)}\gamma T_{Y_I} T_{Y_L} \tag{SB30}$$

$$k_{(4,2)} = \beta_{XY}^{(W)} T_{Y_L} \tag{SB31}$$

$$k_{(5,1)} = \beta_{XY}\chi T_{Y_I} + \beta_{XY}\gamma T_{Y_I} T_{Y_L} \tag{SB32}$$

$$k_{(5,2)} = \beta_{XY} T_{Y_L} \tag{SB33}$$

$$k_{(6,1)} = \beta_{XY}^{(W)}\chi T_{Y_I} + \beta_{XY}^{(W)}\gamma T_{Y_I} T_{Y_L} \tag{SB34}$$

$$k_{(6,2)} = \beta_{XY}^{(W)} T_{Y_L} \tag{SB35}$$

$$\begin{aligned} k_{(1,3)} = {} & \beta_{YX} f_0 f_X \chi T_{X_I^{(0)}} + \beta_{YX} f_0 f_W \chi \sigma_0 T_{X_I^{(0)}} T_{W_I^{(0)}} \\ & + \beta_{YX} f_1 f_X \chi \alpha T_{X_I^{(0)}} T_{X_I^{(1)}} + \beta_{YX} f_0 f_X \gamma T_{X_I^{(0)}} T_{X_L^{(0)}} \end{aligned} \tag{SB36}$$

$$\begin{aligned} k_{(1,4)} = {} & \beta_{YX} f_0 f_X \chi \sigma_0^r T_{W_I^{(0)}} + \beta_{YX} f_0 f_W \chi T_{W_I^{(0)}} \\ & + \beta_{YX} f_1 f_W \chi \alpha T_{W_I^{(0)}} T_{W_I^{(1)}} + \beta_{YX} f_0 f_W \gamma T_{W_I^{(0)}} T_{W_L^{(0)}} \end{aligned} \tag{SB37}$$

$$\begin{aligned} k_{(1,5)} = {} & \beta_{YX} f_1 f_X \chi T_{X_I^{(1)}} + \beta_{YX} f_1 f_W \chi \sigma^1 T_{X_I^{(1)}} T_{W_I^{(1)}} \\ & + \beta_{YX} f_1 f_X \gamma T_{X_I^{(1)}} T_{X_L^{(1)}} \end{aligned} \tag{SB38}$$

$$\begin{aligned} k_{(1,6)} = {} & \beta_{YX} f_1 f_X \chi \sigma_r^1 T_{W_I^{(1)}} T_{X_I^{(1)}} + \beta_{YX} f_1 f_W \chi T_{W_I^{(1)}} \\ & + \beta_{YX} f_1 f_W \gamma T_{X_I^{(1)}} T_{W_L^{(1)}} \end{aligned} \tag{SB39}$$

$$\begin{aligned} k_{(1,7)} = {} & \beta_{YX} f_0 f_X T_{X_L^{(0)}} + \beta_{YX} f_0 f_W \sigma^0 T_{X_L^{(0)}} T_{W_L^{(0)}} \\ & + \beta_{YX} f_1 f_X \alpha T_{W_L^{(0)}} T_{W_L^{(1)}} \end{aligned} \tag{SB40}$$

$$\begin{aligned} k_{(1,8)} = {} & \beta_{YX} f_0 f_X \sigma_r^0 T_{W_L^{(0)}} T_{X_L^{(0)}} + \beta_{YX} f_0 f_W T_{W_L^{(0)}} \\ & + \beta_{YX} f_1 f_W \alpha T_{W_L^{(0)}} T_{W_L^{(1)}} \end{aligned} \tag{SB41}$$

$$k_{(1,9)} = \beta_{YX} f_1 f_X T_{X_L^{(1)}} + \beta_{YX} f_1 f_W \sigma^1 T_{X_L^{(1)}} T_{W_L^{(1)}} \tag{SB42}$$

$$k_{(1,10)} = \beta_{YX} f_1 f_X \sigma_r^1 T_{W_L^{(1)}} T_{X_L^{(1)}} + \beta_{YX} f_1 f_W T_{W_L^{(1)}} \tag{SB43}$$

Finally, after performing the matrix products prescribed in Eq (SB24), it can be shown that the NGM depends only on 8 elements k_{ij} and reads:

$$\mathbf{K} = \begin{bmatrix} 0 & k_{(1,3)} & k_{(1,4)} & k_{(1,5)} & k_{(1,6)} \\ k_{(3,1)} & 0 & 0 & 0 & \\ k_{(4,1)} & 0 & 0 & 0 & \\ k_{(5,1)} & 0 & 0 & 0 & \\ k_{(6,1)} & 0 & 0 & 0 & \end{bmatrix} \tag{SB44}$$

The characteristic equation thus reads:

$$\det(\mathbf{K} - \lambda \mathbb{1}) = -\lambda^3 \left(\lambda^2 - k_{(1,3)}k_{(3,1)} - k_{(1,4)}k_{(4,1)} - k_{(1,5)}k_{(5,1)} - k_{(1,6)}k_{(6,1)}\right) \tag{SB45}$$

which leads to a final closed expression for R_0:

$$R_0 = \sqrt{k_{(1,3)}k_{(3,1)} + k_{(1,4)}k_{(4,1)} + k_{(1,5)}k_{(5,1)} + k_{(1,6)}k_{(6,1)}} \tag{SB46}$$

We use this equation to estimate the value of R_0 for different cities in Madagascar in 2000, when the disease was probably beginning to slowly expand among key population groups embedded in a fully susceptible population (Table SB1 and SB2, see also github site for details on code implementation). In Fig SB1 and Fig SB2, we explore the parameter space in two dimensions (β_Y, p_{YX}). The other parameters values are constant and correspond to the different cities (see Tables SB2 and SB2). Time-dependent parameters F_X, F_Y, δ_X and δ_Y were set to their values in 2000. These figures complement Fig. 3 in the main text. The estimated average female-to-male transmission probabilities (p_{YX}) and the sexual encounter rates (β_Y) are represented by a little circle defining the initial coordinates of the arrow. The tip of the arrow represents the potential reduction in R_0 caused by a 60% reduction in the transmission probability from infectious females to males as a consequence of circumcision (as reported in (15)).

Table SB1. Average model parameter values, and their corresponding values for R_0, and their standard deviations. Average values are calculated over an ensemble of parametric configurations that provide the best model consistency with data under the sigmoidal hypothesis for 5 cities in Madagascar (see Supp Mat for details on R_0 calculation and R_0 values in the other 5 cities)

	**Antananarivo**	**Toliara**	**Taolagnaro**	**Moramanga**	**Nosy Be**
			Disease transmission parameters		
β_Y	108.17 $\pm$ 6.81	107.94 $\pm$ 6.79	107.85 $\pm$ 6.94	107.85 $\pm$ 6.83	107.56 $\pm$ 6.94
β_X	108.24 $\pm$ 6.90	108.12 $\pm$ 6.93	108.33 $\pm$ 6.96	107.79 $\pm$ 6.87	108.08 $\pm$ 6.95
η	4.60 $\pm$ 3.68	11.08 $\pm$ 4.36	16.28 $\pm$ 2.00	13.40 $\pm$ 3.68	9.83 $\pm$ 4.53
m_δ	51.25 $\pm$ 28.43	48.54 $\pm$ 28.45	48.77 $\pm$ 28.81	49.11 $\pm$ 28.70	46.88 $\pm$ 28.29
f_W	0.5349 $\pm$ 0.2851	0.5454 $\pm$ 0.2162	0.3219 $\pm$ 0.1751	0.5649 $\pm$ 0.2817	0.3943 $\pm$ 0.2157
f_0	0.7975 $\pm$ 0.1749	0.6267 $\pm$ 0.2515	0.6401 $\pm$ 0.2253	0.7178 $\pm$ 0.2172	0.4476 $\pm$ 0.2441
p_{XY}	0.0029 $\pm$ 0.0013	0.0027 $\pm$ 0.0014	0.0028 $\pm$ 0.0014	0.0027 $\pm$ 0.0014	0.0028 $\pm$ 0.0014
p_{YX}	0.0005 $\pm$ 0.0003	0.0004 $\pm$ 0.0003	0.0002 $\pm$ 0.0002	0.0005 $\pm$ 0.0003	0.0004 $\pm$ 0.0003
γ	2.2473 $\pm$ 0.9663	2.6933 $\pm$ 0.8075	3.3119 $\pm$ 0.4794	2.5147 $\pm$ 0.8685	2.2787 $\pm$ 0.9820
μ	0.1071 $\pm$ 0.0486	0.1096 $\pm$ 0.0506	0.1322 $\pm$ 0.0529	0.1126 $\pm$ 0.0512	0.1034 $\pm$ 0.0510
χ	53.3454 $\pm$ 21.7434	52.7020 $\pm$ 23.2907	78.3565 $\pm$ 15.6572	63.7132 $\pm$ 22.0065	45.1596 $\pm$ 25.2367
			Demographic parameters		
σ^0	0.0047 $\pm$ 0.0013	0.0212 $\pm$ 0.0066	0.0228 $\pm$ 0.0092	0.0019 $\pm$ 0.0008	0.0301 $\pm$ 0.0097
σ_r^0	0.0240 $\pm$ 0.0166	0.0232 $\pm$ 0.0190	0.0228 $\pm$ 0.0189	0.0265 $\pm$ 0.0160	0.0208 $\pm$ 0.0185
σ^1	0.0004 $\pm$ 0.0005	0.0021 $\pm$ 0.0025	0.0032 $\pm$ 0.0034	0.0004 $\pm$ 0.0003	0.0025 $\pm$ 0.0032
σ_r^1	0.0330 $\pm$ 0.0155	0.0332 $\pm$ 0.0159	0.0335 $\pm$ 0.0163	0.0319 $\pm$ 0.0163	0.0332 $\pm$ 0.0159
α	0.0984 $\pm$ 0.0137	0.1001 $\pm$ 0.0140	0.1008 $\pm$ 0.0141	0.1002 $\pm$ 0.0143	0.0989 $\pm$ 0.0142
A_0	0.0006 $\pm$ 0.0006	0.0035 $\pm$ 0.0028	0.0048 $\pm$ 0.0037	0.0004 $\pm$ 0.0003	0.0051 $\pm$ 0.0043
L_0	1.0984 $\pm$ 0.5620	1.0379 $\pm$ 0.5612	0.9808 $\pm$ 0.5634	1.0175 $\pm$ 0.5370	0.9967 $\pm$ 0.5511
T_0	2011.51 $\pm$ 1.12	2011.45 $\pm$ 1.12	2011.35 $\pm$ 1.12	2011.38 $\pm$ 1.13	2011.44 $\pm$ 1.14
			Basic reproduction number		
R_0	6.580 $\pm$ 3.811	5.865 $\pm$ 1.515	4.428 $\pm$ 0.863	9.389 $\pm$ 4.490	4.967 $\pm$ 1.569

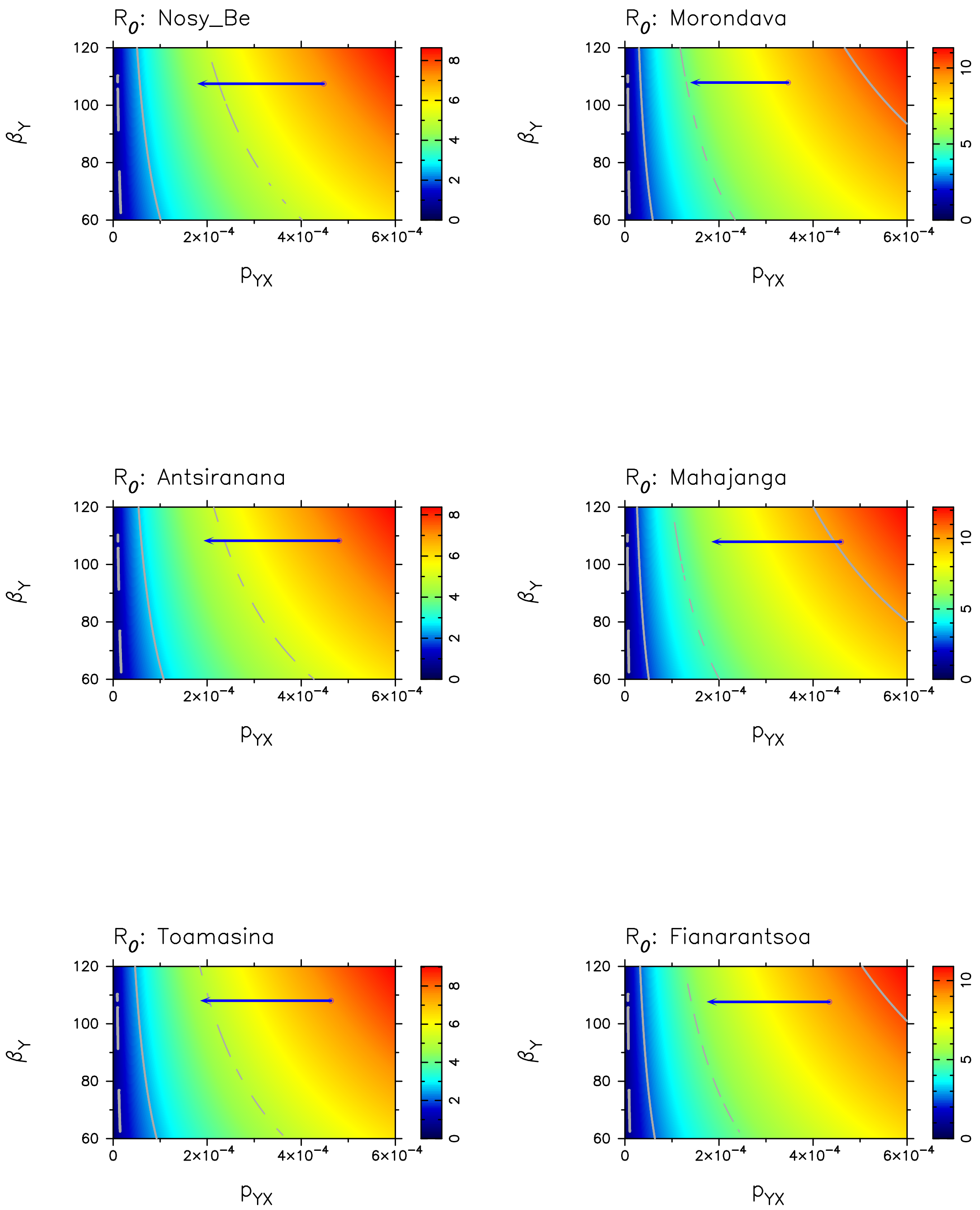

Fig. SB1. Here we show how R_0 (see Eq. SB46) changes as a function of β_Y and p_{YX}. Isoclines of $R_0 = 1.0$ (thick broken lines), $R_0 = 2.5$, $R_0 = 5.0$, and $R_0 = 10$ are also represented. The rest of model parameters, but β_Y and p_{YX}, are kept constant.

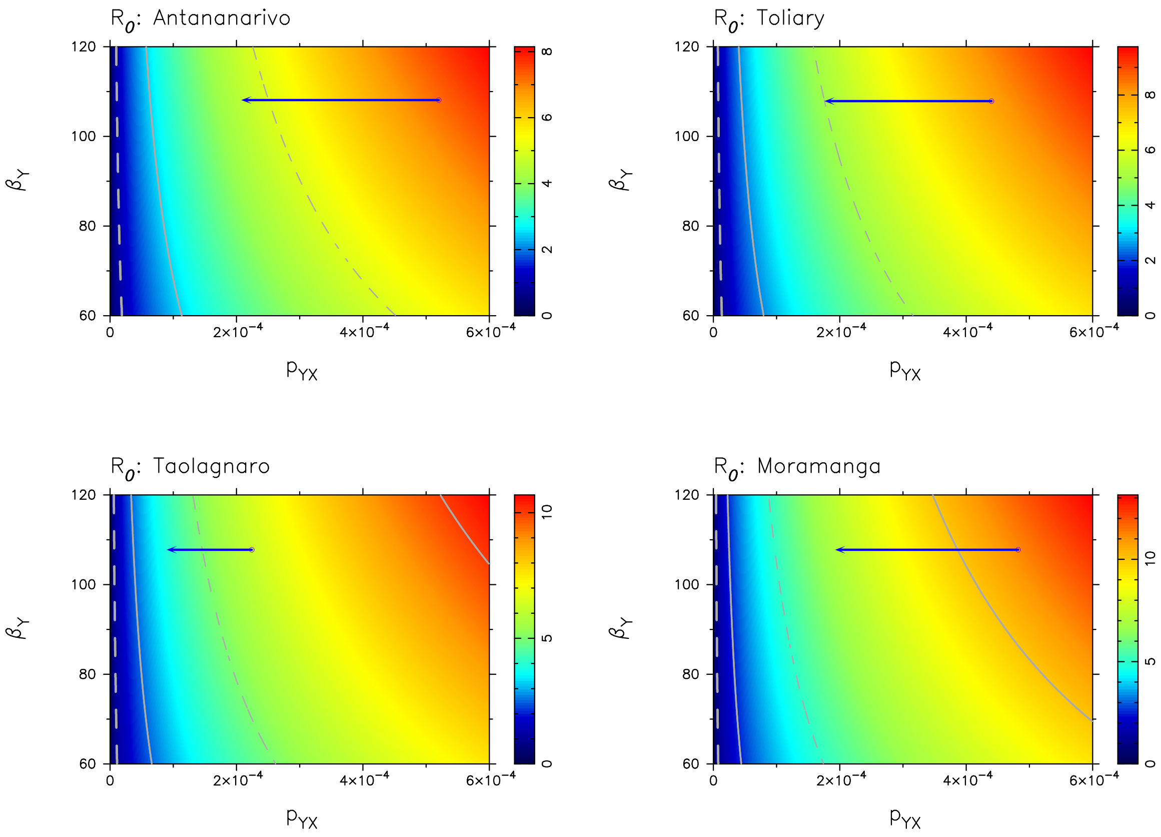

Fig. SB2. Here we show how R_0 (see Eq. SB46) changes as a function of β_Y and p_{YX}. Isoclines of $R_0 = 1.0$ (thick broken lines), $R_0 = 2.5$, $R_0 = 5.0$, and $R_0 = 10$ are also represented. The rest of model parameters, but β_Y and p_{YX}, are kept constant.

Table SB2. Average model parameter values, and their corresponding values for R_0, and their standard deviations.

	**Morondava**	**Antsiranana**	**Mahajanga**	**Toamasina**	**Fianarantsoa**
			Disease transmission parameters		
β_Y	108.00 $\pm$ 6.88	108.35 $\pm$ 6.91	108.00 $\pm$ 7.03	108.13 $\pm$ 7.05	107.70 $\pm$ 6.87
β_X	108.29 $\pm$ 7.00	108.15 $\pm$ 7.08	107.96 $\pm$ 6.92	108.22 $\pm$ 6.89	107.51 $\pm$ 6.99
η	15.97 $\pm$ 2.35	7.78 $\pm$ 4.92	15.91 $\pm$ 2.21	6.20 $\pm$ 4.48	13.98 $\pm$ 4.80
m_δ	47.86 $\pm$ 28.55	49.12 $\pm$ 28.74	39.91 $\pm$ 28.57	51.57 $\pm$ 28.10	51.60 $\pm$ 27.00
f_W	0.4303 $\pm$ 0.2378	0.4866 $\pm$ 0.2074	0.5168 $\pm$ 0.2693	0.4768 $\pm$ 0.2365	0.5272 $\pm$ 0.2500
f_0	0.6399 $\pm$ 0.1966	0.4982 $\pm$ 0.2718	0.5353 $\pm$ 0.1771	0.4556 $\pm$ 0.2444	0.7696 $\pm$ 0.2096
p_{XY}	0.0025 $\pm$ 0.0014	0.0029 $\pm$ 0.0014	0.0028 $\pm$ 0.0013	0.0028 $\pm$ 0.0014	0.0025 $\pm$ 0.0014
p_{YX}	0.0003 $\pm$ 0.0003	0.0005 $\pm$ 0.0004	0.0005 $\pm$ 0.0003	0.0005 $\pm$ 0.0004	0.0004 $\pm$ 0.0003
γ	3.2282 $\pm$ 0.5743	2.0351 $\pm$ 1.0117	3.2528 $\pm$ 0.5239	1.7667 $\pm$ 1.0113	3.0367 $\pm$ 0.6616
μ	0.1241 $\pm$ 0.0514	0.0968 $\pm$ 0.0486	0.1084 $\pm$ 0.0492	0.1012 $\pm$ 0.0497	0.1315 $\pm$ 0.0544
χ	74.72 $\pm$ 17.47	38.35 $\pm$ 25.13	69.04 $\pm$ 19.23	42.62 $\pm$ 26.05	66.88 $\pm$ 29.01
			Demographic parameters		
σ^0	0.0056 $\pm$ 0.0022	0.0419 $\pm$ 0.0122	0.0031 $\pm$ 0.0010	0.0242 $\pm$ 0.0078	0.0059 $\pm$ 0.0023
σ_r^0	0.0228 $\pm$ 0.0169	0.0164 $\pm$ 0.0181	0.0244 $\pm$ 0.0163	0.0235 $\pm$ 0.0187	0.0229 $\pm$ 0.0176
σ^1	0.0008 $\pm$ 0.0009	0.0066 $\pm$ 0.0047	0.0003 $\pm$ 0.0004	0.0023 $\pm$ 0.0028	0.0009 $\pm$ 0.0009
σ_r^1	0.0317 $\pm$ 0.0155	0.0311 $\pm$ 0.0173	0.0317 $\pm$ 0.0150	0.0337 $\pm$ 0.0159	0.0339 $\pm$ 0.0153
α	0.0999 $\pm$ 0.0137	0.0979 $\pm$ 0.0144	0.0992 $\pm$ 0.0139	0.0998 $\pm$ 0.0141	0.1000 $\pm$ 0.0141
A_0	0.0010 $\pm$ 0.0009	0.0112 $\pm$ 0.0082	0.0004 $\pm$ 0.0004	0.0039 $\pm$ 0.0030	0.0011 $\pm$ 0.0009
L_0	1.0277 $\pm$ 0.5518	0.9836 $\pm$ 0.5757	1.0663 $\pm$ 0.5468	1.0622 $\pm$ 0.5666	1.0052 $\pm$ 0.5485
T_0	2011.43 $\pm$ 1.14	2011.31 $\pm$ 1.18	2011.49 $\pm$ 1.13	2011.45 $\pm$ 1.11	2011.41 $\pm$ 1.14
			Basic reproduction number		
R_0	6.411 $\pm$ 2.247	4.962 $\pm$ 1.538	8.612 $\pm$ 3.781	5.272 $\pm$ 2.001	6.302 $\pm$ 2.268

C. Parameter estimation from demographic data

In order to add realism to our projections, we considered real Madagascar demographic data from governmental and institutional sources (US government, the World Health Organization, and Institut National de la Statistique, Antananarivo (16–18))

For each year, between 2000 and 2016, four parameters were first estimated directly from data: annual *per capita* mortality rates for males and females (δ_X and δ_Y), and total number of males (F_Y) and females (F_X) reaching sexual maturity every year. Their values are chosen to capture the temporal evolution of urban adult populations in the most important cities of Madagascar (see also Table F1). Since we only had demographic data at the national level, no further differences between cities were considered. Age-dependent *per capita* average rates were assumed the same across the eleven cities under study.

In this section, we first give further details on the estimation of annual mortality rates, δ_X and δ_Y, and recruitment rates, F_X and F_Y. Once these four demographic parameters are fully estimated for every city, we use the available information of the distribution of females in the different groups to obtain parameter values for the five rates (σ^0, σ^0_r, σ^1, and σ^1_r, and α), which are responsible for the distribution of females into young and adult, non-sexual and sexual worker groups. The few available data that exist show that this distribution was different in every city and changed considerably from 2014 to 2017 (see Table F1).

A. Fecundity and mortality rates. Here we outline the strategy for δ_X, δ_Y, F_X, and F_Y parameter estimation over time. F_X and F_Y depend on female overall fertility from previous years, which in turn, depends on the local reproductive population at that point in the past. This would require the use of delayed ODEs models. The SICA model gets around this complexity by estimating F_X and F_Y directly from demographic table data. In this sense, F_X, F_Y, δ_X, and δ_Y become data-derived parameters with empirical trends and year-to-year variability. By summing up equations for males and females in the full system, it is easy to show that in the absence of disease population dynamics in SICA model depend only on two sex-specific recruitment rates, F_X and F_Y, and two mortality rates, δ_X and δ_Y. Demography therefore can be simply be represented in a two-equation system:

$$\begin{aligned} \frac{dY}{dt} &= F_Y - \delta_Y\, Y \\ \frac{dX}{dt} &= F_X - \delta_X\, X \end{aligned} \qquad \text{[SC1]}$$

This is a non-age-structured model tracking the temporal evolution of female and male total adult populations. However, both fertility—which determines recruitment rates F_X and F_Y— and mortality are age-specific processes. Despite this simplified demographic assumption, we can reasonably estimate non-age-specific model rates from age-specific data, and thus capture year-to-year realistic population variability and average trends.

A.1. Average mortality rates. Human mortality is clearly an age-dependent process. However, the SICA model assumes that mortality rates are constant, rather than age-dependent. Namely, it is assumed that survival curves are negative exponentials, which means that individual expected life-span can be represented by the inverse of a mortality rate $1/\delta$. A reasonable way to take into account year-to-year variability and a slight sex-dependency in adult mortality rates is then using the life-expectancy at the age of entrance into adult life L_A. As an estimate of a time-dependent $1/\delta$ parameter, we used compiled life-table data to extract the life expectancy for every year of the study period (2000-2016) of the age group 15-19. Specifically, we consider:

$$\begin{aligned} \delta_Y(t) &= \frac{1}{L_A^Y(t)} \\ \delta_X(t) &= \frac{1}{L_A^X(t)} \end{aligned} \qquad \text{[SC2]}$$

where $L_A^Y(t)$ and $L_A^X(t)$ are the life table life-expectancy values in the 15-19 age group for males and females, respectively, at year t (from 2000 to 2016).

A.2. Recruitment Rates. The F_Y and F_X parameters of the SICA model represent the number of males and females, respectively, entering a fully active sexual life per unit time. In general, these rates have year-to-year variability. Let a_Y and a_X be the starting age for active sexual life for males and females, respectively. If we knew the age distribution every year, the number of males and females passing from age $a_X - 1$ to a_X, and from $a_Y - 1$ to a_Y, respectively, in year t would be directly the empirical time-dependent parameters $F_X(t)$ and $F_Y(t)$ that the SICA model requires. However, such a degree of detail is very difficult to gather in demographic studies. In the absence of this detailed demographic data, F_Y and F_X at time t can instead be estimated from the total number of males and women born in year $t - a_Y$ and $t - a_X$, respectively, along with sex- and age-specific mortality rates. Fortunately, such data have been regularly compiled and made publicly available by the WHO and other institutions at least on a quinquennial basis for every country in the world.

Let $B_Y(t)$ and $B_X(t)$ be the total number of males and females born in year t. As we show below, these two numbers can be in turn estimated from average *per capita* total fertility and total populations in a given year t. Since the two cohorts $B_Y(t - a_Y)$ and $B_X(t - a_X)$ will suffer from age- and sex-dependent mortality up to year t, the parameters $F_Y(t)$ and $F_X(t)$

can be estimated by calculating the number of survivors until year t for those boys and girls born in years $t - a_Y$ and $t - a_X$, respectively. Therefore, the parameters of interest can be estimated as:

$$
\begin{aligned}
F_X(t) &= B_X(t - a_X)\, s_X^{(0)}(t - a_X)\, s_X^{(1)}(t - a_X + 1) \ldots s_X^{(a_X - 1)}(t - 1) \\
F_Y(t) &= B_Y(t - a_Y)\, s_Y^{(0)}(t - a_Y)\, s_Y^{(1)}(t - a_Y + 1) \ldots s_Y^{(a_Y - 1)}(t - 1)
\end{aligned}
\qquad \text{[SC3]}
$$

where $s_X^{(a)}(t)$ is the survival probability during year t of female individuals of age a. For instance, $s_X^{(0)}(t)$ is survival probability of new-born females during year t; those who will on average have their one-year birthdays in year $t+1$.

Eqs. (SC3) show that the estimation of the recruitment rates F_Y and F_X requires estimating, first, survival probabilities and, second, the number of new-born individuals for a given year. Let us proceed in order.

Survival Probabilities

In typical life tables, age-dependent mortality rates are associated to finite time intervals of a given number of years n. They are defined as:

$$m_a^n(t) \equiv \frac{D_a^n(t)}{\langle N_a^n(t) \rangle} \qquad \text{[SC4]}$$

where $D_a^n(t)$ is the number of deaths in the age group between age a and age $a+n-1$ during year t, and $\langle N_a^n(t)\rangle$ is the mid-year population in that age group at year t. Survival probabilities can be estimated from this statistic under the assumption of constant mortality within each age group. The survivors at the end of a year are related to the initial number of individuals at the beginning of that year for each age group through an exponential decay:

$$N_a^n(t+1) = N_a^n(t) \exp(-\delta_a) \qquad \text{[SC5]}$$

where δ_a is an age-specific instantaneous mortality rate assumed constant for all individuals within the same age group, which means, individuals between age a and age $a+n-1$ experience the same probability of death. In addition, $\langle N_a^n(t)\rangle$ can be approximated by an average population in that age group:

$$\langle N_a^n(t) \rangle = \frac{N_a^n(t+1) + N_a^n(t)}{2} \qquad \text{[SC6]}$$

By introducing Eqs (SC5) and (SC6) into Eq. (SC4), the age-dependent mortality rate of age group a at year t, can be rewritten as:

$$\frac{m_a^n(t)}{2} = \frac{1 - \exp(-\delta_a)}{1 + \exp(-\delta_a)} \qquad \text{[SC7]}$$

By inverting the last equation, instantaneous time-dependent mortality rates, δ_a, corresponding to each age group a and year t can be estimated from the empirical age-dependent mortality rate values, $m_a^n(t)$, which are typically given in demographic studies. The inversion of Eq (SC7) can be written as:

$$\delta_a(t) = \ln\left(\frac{1 + m_a^n(t)/2}{1 - m_a^n(t)/2}\right) \qquad \text{[SC8]}$$

Once instantaneous death rates are estimated for each sex, year, and age group, all survival probabilities appearing in Eq (SC3) can be also estimated. Notice that a survival probability for a one-year time interval is defined as:

$$s^{(a)}(t) \equiv \frac{N_a^n(t+1)}{N_a^n(t)} \qquad \text{[SC9]}$$

which, using Eq. (SC5), leads to:

$$s^{(a)}(t) = \exp(-\delta_a(t)) \qquad \text{[SC10]}$$

where the year-to-year dependency of $\delta_a(t)$ is here explicitly written (see Eq. (SC8)).

Number of Newborns at Year t

All that is now left to estimate are the quantities $B_Y(t)$ and $B_X(t)$, the number of male and female new-borns in the population at year t. Again detailed demographic population monitoring would ideally register these two quantities every year. In the absence of this direct information, we can estimate these values from total fertility rates, total female population, and sex ratio at birth:

$$
\begin{aligned}
B_X(t) &= (1 - f)\, \Phi(t)\, X(t) \\
B_Y(t) &= f\, \Phi(t)\, X(t)
\end{aligned}
\qquad \text{[SC11]}
$$

where f is the ratio of males to females (*sex ratio* at birth), $\Phi(t)$ is an average *per capita* fertility rate (per year), i.e. the number of children an average female has per unit time, and $X(t)$ is the total number of females in the population at time t.

For instance, Madagascar sex ratio at birth is about 1.02, while adult sex ratio is about 1.0. The average fertility rate $\Phi(t)$ can be estimated from the total fertility rate $b(t)$, i.e. the average number of children born to an average female during her whole reproductive life if she follows the birthing pattern being experienced by the overall population at year t.

Assuming that female reproductive age spans from age $a_X^{(0)}$ up to $a_X^{(1)}$, both rates can be estimated from total annual births stratified by age and female population age distribution according to the following definitions:

$$\Phi(t) = \frac{\sum_{j=a_X^{(0)}}^{a_X^{(1)}} B^{(j)}(t)}{X(t)} \quad \text{[SC12]}$$

$$b(t) = \sum_{j=a_X^{(0)}}^{a_X^{(1)}} \frac{B^{(j)}(t)}{X^{(j)}(t)} \quad \text{[SC13]}$$

where $B^{(j)}(t)$ is the annual number of births from females at specific age j. We can realistically assume that the age, a_X, of entrance to active sexual life matches $a_X^{(0)}$. Notice also that $\Phi(t)$ and $b(t)$ are not expressed in the same units. While $\Phi(t)$ is a *per capita* annual rate, $b(t)$ is the female *per capita* total fertility during her whole fertile period, which is between ages $a_X^{(0)}$ and $a_X^{(1)}$. We can express $b(t)$ as a *per year* rate by dividing it by $(a_X^{(1)} - a_X^{(0)})$. However, the relation between these two rates is not quite evident. Since total fertility $b(t)$ is the one usually provided by typical demographic surveys, we estimate the annual *per capita* fertility rate $\Phi(t)$ from the *per capita* total fertility $b(t)$ in the following way:

$$\Phi(t) = r(t)\,\frac{b(t)}{(a_X^{(1)} - a_X^{(0)})} \quad \text{[SC14]}$$

where $r(t)$ is a time dependent parameter that could be estimated from data by using the previously given definitions in Eqs (SC12) and (SC13) to empirically fulfill Eq (SC14). For our purposes, we have made the simplifying assumption that the parameter $r(t)$ is roughly constant through time.

In summary, the SICA model parameters, F_X and F_Y, have been estimated from demographic table data by the following equations:

$$F_X(t) = (1-f)\,r\,\frac{b(t-a_X^{(0)})}{(a_X^{(1)} - a_X^{(0)})}\,\frac{N(t-a_X^{(0)})}{2}\,s_X^{(0)}(t-a_X^{(0)})\,s_X^{(1)}(t-a_X^{(0)}+1)\ldots s_X^{(a_X^{(0)}-1)}(t-1)$$

$$F_Y(t) = f\,r\,\frac{b(t-a_Y^{(0)})}{(a_X^{(1)} - a_X^{(0)})}\,\frac{N(t-a_Y^{(0)})}{2}\,s_Y^{(0)}(t-a_Y^{(0)})\,s_Y^{(1)}(t-a_Y^{(0)}+1)\ldots s_Y^{(a_Y^{(0)}-1)}(t-1)$$

where $N(t)$ is the adult population at year t, f is the sex ratio at birth, and r is a constant free parameter. Since we know the sex ratio is balanced in Madagascar, for our estimates we calculated female population $X(t)$, by dividing the total adult population $N(t)$ by two.

In theory, according to Eqs. (SC15)-(SC15), r is a correcting factor to account for the effective female population contributing to local births every year. In practice, r should be regarded as a free model parameter used to scale the population temporal evolution to a realistic average level, and approximate the population trajectory for every city in an optimal fashion. This is achieved through a simple fitting procedure. Heuristically, the r parameter could potentially account for some extra population growth due to immigration from rural areas.

In sum, recruitment rates F_X and F_Y should be interpreted as two functions of time t, accounting for the reproductive output of the population at some previous year $t - a_X$, weighted by the total survival probability until present time — see Eqs (SC15)-(SC15). This introduces a time delay in our dynamical equations. WE have shown that we can get around the complexity of delayed ODEs, and make the simple ODE system given in Eqs. (SC1) best match observed population trajectories. In order to do that, we have approximated female reproductive population by a fraction of total observed population at any given year. In theory, since sex ratio is balanced, female population is simply 0.5 times total adult population. We would expect this r fraction to be related to the relative importance of non-local births to the local recruitment. More precisely, total female recruitment is a compound quantity including both locally born $F_X^{(0)}$, and immigrated individuals $F_X^{(i)}$ per year:

$$F_X = F_X^{(i)} + F_X^{(0)} \quad \text{[SC15]}$$

Although our fitting method is not devised to estimate these two quantities independently, r values encode information about a non-negligible effect of immigrated women on the local growth of the cities. Our empirical estimates of total F_X and F_Y (with $r > 0.5$) suggest this effect. In Fig SC1, we present the output of the demographic model and total population data from Antananarivo for comparison. In this case, we found $r = 0.615$.

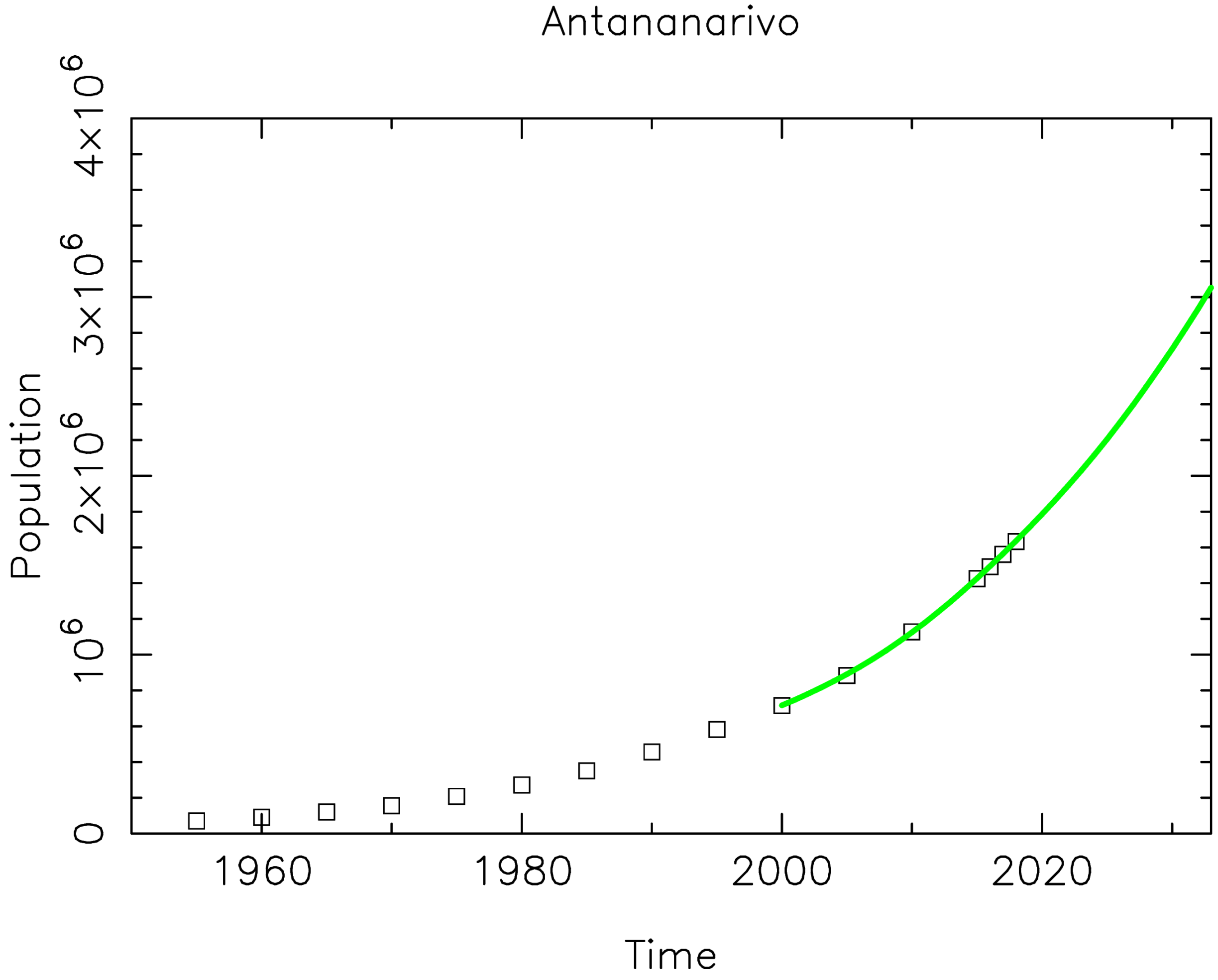

Fig. SC1. Demographic trends in Antananarivo. The numerical integration of the demographic model in Eqs (SC1), taking the population in 2000 as the initial condition, and time-dependent parameters F_Y, F_X, δ_X, δ_Y, all estimated from data from 2000 to 2016, produces the solid green line. Squares represent population data from source: `http://www.worldometers.info/world-population/madagascar-population/`. These data were used for model parameter estimation.

B. The female population distribution. Unfortunately, time series data on the actual female distribution across the four groups considered in every city were not available. We only had the sex worker population (per city) in 2014 and 2017 (see Table F1). Since the relative distribution across groups is determined by the relation of in-rates to out-rates, both very high and very low rate values (or anything in between) can be adjusted to yield the same fraction of females in each category at stationarity. We therefore had to make an extra, but reasonable, assumption: we assumed that these rates should be rather slow (see adopted range values in Table SA1), which means that sexual habits tend to change at the scale of years rather than weeks or months. For instance, if a young female decides to increase her sexual contact rate for transactional sex reasons, she will remain in these highly active sexual phase for at least a couple of years. As we explained in the main text, throughout this work we considered the sex-working stage *sensu lato*, although sex working population surveys are probably not able to uncover transactional sex, which means that our predictions might be quite conservative. Females in this sexually highly active stage are characterized by an up to 10-fold relative increase in their sexual contact rates (see parameter η in Table SA1 and Tables SB2 and SB1). In practice, these higher contacts mainly result from casual transactional sex rather than from professional sex work.

Average ages at which males and females are considered fully sexually active were chosen to be at 15 and 17 years of age, respectively. The *aging* rate α is not a typical demographic parameter, but rather defines the average time females are considered *young* by males in terms of assortative sexual contacts or sexual preference. In our projections and parameter searches, this parameter was considered to be between 8 and 12 year^{-1} ($1/\alpha \approx 0.1$, see Table SA1 and Tables SB2 and SB1). That is, females are *young* as long as they are about between 15 and 23 to 27 (average 25) years old. There is an undetermined variance around all these parameter values, with no exception, which reflects individual heterogeneity. Although probably important, the consideration of this extra complexity is beyond the scope of the modeling approach presented here.

Parameter estimation relies on the definition of a likelihood function addressing the question: what is the probability of observing a given time series of certain population and epidemiological variables if one assumes that disease transmission dynamics is generated by our model structure, as defined by an ODE system, with a given set of parameter values, this is, a given parametric configuration? Further details on parameter estimation strategies are given in the following section.

D. Model Validation

This process was done in three different phases: the assessment and validation of the simple demographic model (Eqs SA13), the expanded demographic model (Eqs SA12), and finally, the disease transmission model (Eqs (SA1)-(SA8)). We define first a generic likelihood function, and then the three particular likelihood functions employed, and report further details on these three steps in order.

A. The likelihood function. Let $\mathbf{X^{(o)}}$ be a matrix of observed data for $t = t_0$ to $t = t_n$. The matrix is built by row vectors, each of them corresponding to a time series of observed variables, from $\vec{X}_1^{(o)}$ to $\vec{X}_N^{(o)}$:

$$\mathbf{X^{(o)}} \quad = \quad \begin{bmatrix} X_1(t_0) & \cdots & \cdots & X_1(t_n) \\ \cdots & \cdots & \cdots & \cdots \\ X_N(t_0) & \cdots & \cdots & X_N(t_n) \end{bmatrix}$$

Observed variables can be related to model variables via output variables, i.e. variables that are a given mathematical function of system state variables. For instance, total prevalence within the SW population is one of these output variables. For each observed variable we will thus have a predicted output variable from model numerical integration. In this situation, we can ask: "What is the probability of observing this data matrix under the assumption that our model and its specifying parameters are true?". This is:

$$P(\vec{X}_i^{(o)}|\theta,\theta_e,\epsilon) = \prod_{j=0}^{n} P(X_i^{(o)}(t_j) - X_i(t_j)|\theta,\theta_r,\epsilon)$$

$$P(\mathbf{X^{(o)}}|\theta,\theta_e,\epsilon) = \prod_{i=0}^{N} P(\vec{X}_i^{(o)}|\theta,\theta_e,\epsilon)$$

This likelihood function relies on the error functions, or probability of obtaining certain deviations from predicted values, $E_i(t_j) \equiv X_i^{(o)}(t_j) - X_i(t_j)$ given our model structure and parameters values:

$$P(X_i^{(o)}(t_j) - X_i(t_j)|\theta,\theta_r,\epsilon) = P(E_i(t_j)|X_i(t_j),\theta,\theta_e,\epsilon) \qquad \text{[SD1]}$$

where θ should be regarded as a full model parameter set (a model parametric configuration), θ_r are parameters controlling the error function, e.g. variances, and ϵ is an additional parameter to accurately calculate probabilities from their corresponding continuous density functions:

$$P(E_i(t_j)|X_i(t_j),\theta,\theta_e,\epsilon) = \int_{E_i(t_j)-\epsilon}^{E_i(t_j)+\epsilon} f_E(x|X_i(t_j),\theta,\theta_e)dx \qquad \text{[SD2]}$$

Although we have tried several error choices (results not shown), throughout this work, our results rely on Gaussian errors with varying variances, this is:

$$P(X_i^{(o)}(t_j)|X_i(t_j),\theta,\sigma,\epsilon) = \frac{1}{\sigma\sqrt{2\pi}}\int_{X_i^{(o)}(t_j)-\epsilon}^{X_i^{(o)}(t_j)+\epsilon} e^{\frac{1}{2}\left(\frac{x-X_i(t_j)}{\sigma}\right)^2} dx \qquad \text{[SD3]}$$

where σ is the error standard deviation which may depend on t_j. Parameter distributions were obtained by minimizing the negative loglikelihood (see Eq (SD1)), and filtering those that were only 2 points apart from the optimal one in their negative loglikelihood values. This is a typical specification on likelihood ratio tests. Parametric configurations producing loglikelihoods more than 2 units farther apart from the optimal one make data *significantly* less likely (0.01 times less likely) than the likelihood of the optimal one.

B. The simple demographic model. We can summarize F_X and F_Y in Eqs (SC15)-(SC15), mathematically, as:

$$F_X = F_X(t,r|\mathbf{D})$$
$$F_Y = F_Y(t,r|\mathbf{D})$$

where r is a free parameters and $\mathbf{D}$ is the full set of demographic data, stored as yearly demographic tables, namely sex ratio f, ages $a_X^{(0)}$ and $a_X^{(1)}$, male and female life expectancy, age-dependent mortality rates, total fertility rates, and observed total populations for each year t. Although we write these functions for convenience here with an explicit time-dependence, this is implicit in the year-to-year variability of the different parameters, appearing in Eqs. (SC15)-(SC15), and directly estimated from annual demographic tables.

For each city, we know the real population trajectory $N_o(t)$, from $t = t_0$ to t_1. Note the subscript o stands for *observed*. Through simple numerical integration of the ODE system (SC1), we can generate a model prediction for that trajectory and a given value of r and $N(t,r)$ (from t_0 to t_1), where $N^{(o)}(t_0)$ is taken as the initial condition. The best estimate of the free parameter r is then determined using a Gaussian error function, in particular as a measure of the difference between empirical and model predicted population values. Assuming independent errors, and a constant variance, a likelihood function describes the probability of observing certain reported time series $\mathbf{D} = (N^{(o)}(t_0),\ldots,N_o(t_1))$, on total population increase in Madagascar:

$$P(\mathbf{D}|r) = \prod_{t=t_0}^{t_1} P(N^{(o)}(t) - N(t,r)|r,\epsilon,\theta_e) \qquad \text{[SD4]}$$

where θ_e are the parameters controlling the distribution of errors, for instance, the variance for Gaussian errors. In addition, an extra parameter ϵ was used (with value of 2.0) to be able to deal with continuous random variables, governed by continuous density functions. Each factor, i.e. the probability of observing $N^{(o)}(t)$ individuals at time t, should be properly written as $P\left[N(t,r)-\epsilon \;<\; N^{(o)}(t) \;<\; N(t,r)+\epsilon\middle|\,\theta\right]$ (see Eq (SD3)).

As usual, the optimization procedure minimizes the negative loglikelihood function. An error function with non-time-dependent variance implies the minimization of the sum of squared deviations from deterministic predicted values obtained through the numerical integration of the dynamical system. We have instead presented the full likelihood function here because we made use of other error functions and explored an assumption about the error variance. In particular, we made the assumption that the error variance of our population measures is proportional to the expected value $N(t,r)$. In any case, our resulting projections were robust to different assumptions on the error function of choice.

In our analysis, we included reliable estimates of urban populations for every city in 2013, data on the temporal evolution of total urban population in Madagascar, and life table data. The sum of the populations of the 11 cities represented only a fraction of the estimated total urban population. We assumed that this fraction remained roughly constant over the whole period. We then calculated the fraction of the population in 2013 for each and every of the 11 cities. We assumed that this share among the cities remained also constant over the whole period. Finally, we had reliable estimates of the fraction of children (between 0 and 15 years) in the total Madagascar population, in 1975 (44.4 %), 2017 (39.87 %), and 2018 (39.55 %) (19). We used this data to interpolate adult fractions over the period of interest (2000-2016). These assumptions combined allowed to estimate, yearly and empirically, adult populations for every city from 2000 to 2016. Given the high quality of life table data, these estimates are very reliable. By using the likelihood function above, we compared model predictions for temporal trajectories of the adult population in every city with the obtained empirical estimates of those from demographic data.

In sum, demographic data from Madagascar were accurate enough to successfully estimate model demographic parameters (recruitment F_X, F_Y, and mortality rates, δ_X and δ_Y) directly from demographic tables and fecundity data. In addition, we required to fit an extra constant parameter r. This parameter can be regarded as a correction factor on recruitment rates to exactly match observed population increase in every city. It can be also interpreted as a measure of the influence of immigration from rural areas into the city on the number of females and males entering active sexual life per year. If this number is greater than 0.5, there is a positive immigration. If it is lower than 0.5, it means that there is a loss of children that were born in the city but emigrated out from the it before reaching sexual maturity. We obtained an r value of 0.615. These parameter estimates, both the time-dependent F_X, F_Y, δ_X, δ_Y, and the constant r parameters, produced model trajectories for the temporal evolution of the adult population for each of the cities in extremely good agreement with empirical data (see Fig SD1 and SE3).

Table SD1. Different specifications of the sigmoidal hypothesis were tested. From H_1 to H_9, we constrained pseudo-data generation to match the number of sexual workers observed in 2017. H_7 to H_9 represent quite an abrupt increase around the threshold year. They produced poor fits, this is, lower levels of consistency between available data and our expanded demographic model, and were not further considered. An extra sigmoidal hypothesis (H_0), identical to H_5, but assuming the number of sex workers as those observed in 2014, was also tested.

		T_0		
		2010	2011	2012
L_0	0.01	H_1	H_2	H_3
	0.1	H_4	H_5	H_6
	1	H_7	H_8	H_9

C. The expanded demographic model. In a second step, we used the data of the sex-working population per city (see data from Table F1) to obtain estimates of the parameters that control the temporal evolution of the distribution of females in the four classes considered (sex, non-sex worker, and young and old groups). Note that we only had data of the sex-working population for each city in 2014 and 2017 (see Table F1). These two values showed large error bars and, for some cities, were not fully consistent with each other. This may be a consequence of the inherent difficulties associated to reliably censusing these groups. We should admit that these limitations introduce uncertainty in our model predictions.

However, in spite of the scarcity of temporal data points, in order to find plausible parameter combinations, model parameters were searched to fit a trajectory for the number of sex workers that could have been observed from 2000 to 2016 (see Fig SD1, panel B). These *pseudo*-data were generated under two reasonable hypotheses, namely, the *constant-fraction* and the *sigmoidal* hypotheses. The first one assumes that the different values observed in 2014 and 2017 represent natural fluctuations of the fractions of sex workers with respect to the total women adult population around an average level. This hypothesis assumes this average fraction is constant over the whole period of interest (2000-2016). The second hypothesis takes into consideration Madagascar economic crises (2009-2013) and makes the assumption that the fraction of sex workers within the adult female population could have increased. We modeled this growth as a sigmoidal curve. This involves assuming lower fractions of sex workers in 2000, and fractions of SW approximately matching the truly observed number by the end of the period. This requirement fixes one of the the three parameter of the sigmoidal curves, which was chosen to be A_0 (see Eqs. (SD5)-(SD6)). Therefore, in order to fully specify this hypothesis, two extra quantitative assumptions are still required: the smoothness of the sigmoidal jump (L_0) and the threshold value (T_0) in Eqs. (SD5)-(SD6)). We characterized our curves by a smoothness parameter of about 0.1 (in a range from 0.01 to 1.0), and a threshold value around 2011 (in a range from 2009 to 2013). We explore a total of 10 parametric combinations (see Table SD1).

In sum, both hypotheses assumed lower absolute numbers of SW at the beginning of the period, which would have increased either only due to population expansion (*constant-fraction* hypothesis), or in addition, sigmoidally as a consequence of the 2009-2013 economic crisis (*sigmoidal* hypothesis). Therefore, in our parameter searches, we had then three observed variables in every city:

- Total adult male population
- Total adult female population
- Total sex worker population (as created according to either the *constant-fraction* or the *sigmoidal* hypothesis).

By specifying the likelihood function (see (Eq SD1,) we obtained parametric configurations for each of the hypotheses. While under the *constant-fraction* hypothesis, σ_0, σ_1, σ_0^r and σ_1^r were all considered constant in time, under the *sigmoidal* hypothesis, we consistently let σ_0, and σ_1 parameters evolve in time following also a sigmoidal curve (see Fig SD1, panel D), but we still kept σ_0^r and σ_1^r constant. Sigmoidal curves are flexible enough to represent a sudden increase in a quantity after a certain threshold is crossed. In our case, before the threshold year, sex workers were assumed to be in lower fractions. After that year, more and more women might have used transactional sex to cope with harsh economic conditions. In particular, we consider the following sigmoidal curves:

$$\sigma_0(t) = \sigma_0 + \frac{A_0}{1+e^{-L_0\,(t-T_0)}} \quad \text{[SD5]}$$

$$\sigma_1(t) = \sigma_1 + \frac{A_0}{1+e^{-L_0\,(t-T_0)}} \quad \text{[SD6]}$$

In any case, α and the four σ parameters, and the three governing the two sigmoidals (see Eqs. (SD5)-(SD6) above), under the second hypothesis, were all estimated to fit plausible time series for the number of sex workers within the adult female population and the total adult female and male city populations observed during the studied period (2000-2016). We did this for the 11 eleven cities in Table SF1. In Fig SD1 we show only results for the *sigmoidal* hypothesis in Antsiranana. In bottom right panel, the sigmoidal time dependence in one of the σ parameters is shown across the parameter distribution that best fitted the data .

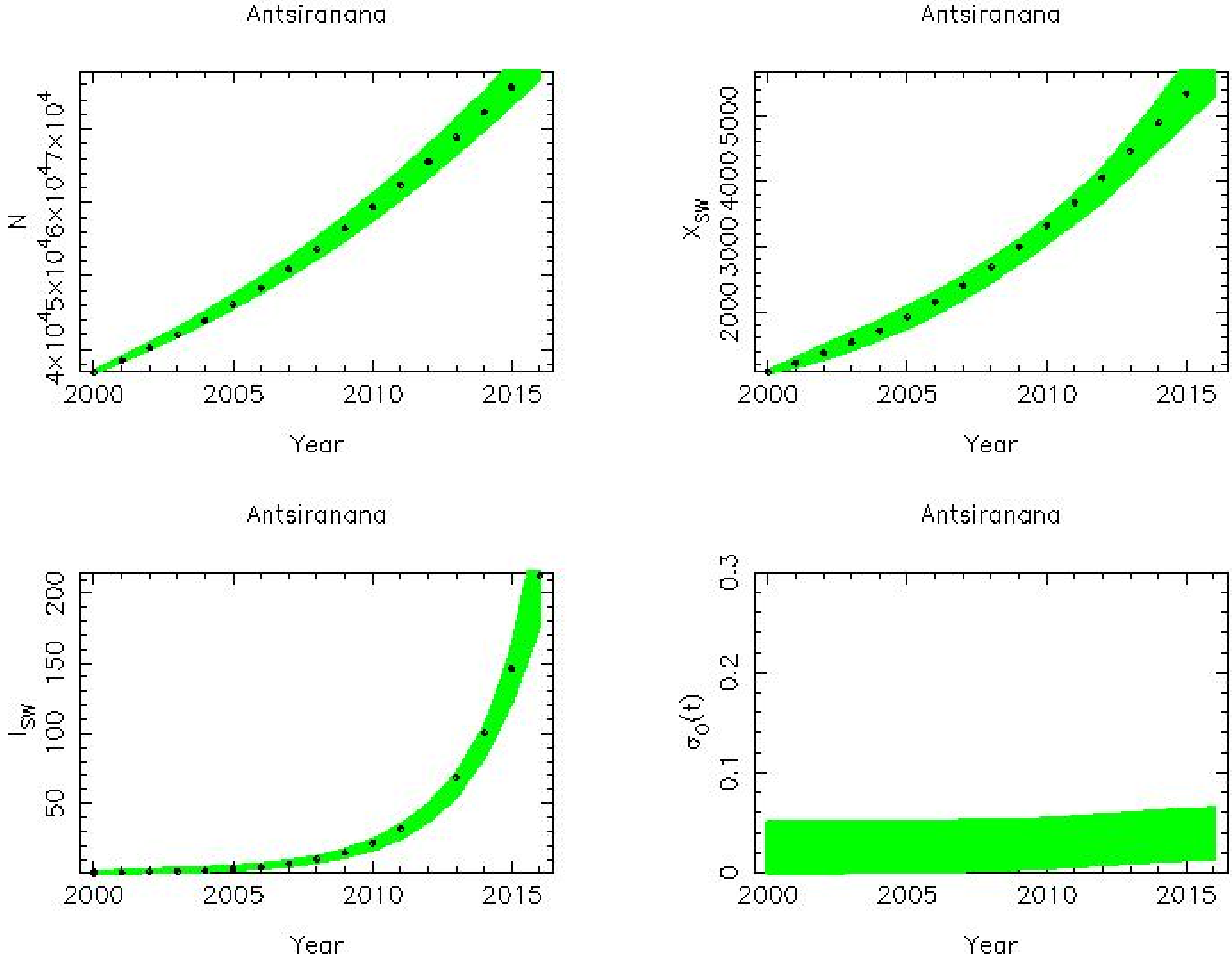

Fig. SD1. Demographic trends in Antsiranana. The numerical integration of the demographic model in Eqs (SC1), taking the population in 2000 as the initial condition, and the four time-dependent parameters F_Y, F_X, δ_X, δ_Y, and a extra time-constant parameter r estimated from data from 2000 to 2016, predicts the solid green line representing the temporal evolution of total adult population (Panel A). Circles represent yearly interpolated values from demographic empirical data. Population data from source: `http://www.worldometers.info/world-population/madagascar-population/`. The temporal evolution of the total number and the number of infected individuals of the sexual working female population is plotted in panels B and C, respectively, for a number of parameter combinations each of them predicting a similar time trajectory (different green lines). In panel B, single circles correspond to data generated under the hypothesis of a sex-working population increasing as a sigmoidal curve with a threshold parameter at year 2011, smoothness parameter 0.1, and 2014 SW levels. In panel D, we also show the temporal evolution of σ_0 associated to the same ensemble of parameter configurations producing good fits.

D. The full disease-transmission model. Finally, we use data of 2016 on HIV prevalence across cities within the sex-working (SW) population (see Table SF1) to search for parameter combinations able to produce trajectories compatible with the data at hand. Predicted trajectories result from numerical integration of the full ODE system. We left out the observed data on HIV prevalence of previous years. Although prevalence data (within the sexual working, SW, population) for some cities does exist for 2005, 2007, and 2010, and 2012 (see Table SF2), years before 2016 all reveal very low levels (less than 2.5%). It is not until 2016 that disease prevalence remarkably increases in most cities reaching values over 10% in some of them. Therefore, we left out previous years for visual validation purposes (see Fig SE4, SE5, and SE6).

Here we assumed that HIV has been slowly expanding for the last decades in Madagascar until reaching observed levels in 2016. This is in full agreement with public health information and sparse, but valuable data from HIV-AIDS in Madagascar (20). The initial phase of an infectious disease is always exponential in a very good approximation. This is a common feature of the initial expansion of any infectious disease (2). Therefore, this hypothesis involves an exponential expansion of disease prevalence from very low values around 2000 to the observed 2016 levels. Unlike the previous constant-fraction *vs* sigmoidal hypotheses, this exponential expansion can be better regarded as an empirical fact than as a true hypothesis given that all information about AIDS in Madagascar points to a slow but steady expansion of the disease in the island for the last decades. In any case, the full characterization of this expansion involves an extra quantitative assumption: we need to prescribe SW disease prevalence in 2000. We considered only 1% of that observed in 2016. Given prevalence values and population numbers for SW, this percentage implies that only a few SW individuals were already infected (lower than 10 individuals) at that initial year. Then disease prevalence within SWs would have increased exponentially from that initial low level, at the beginning of the studied period, up to 2016 levels.

We could have let the initial condition be defined by parameters to be searched as well. However, the beginning of the studied period is supposed to be quite well defined by very low levels of disease incidence in key exposed groups (SW) and neglishable levels within the general population (20). Just in case, we explored different prevalence levels at the beginning of the studied period (1%, 5%, 25%, and 50% of the prevalence levels observed by the end the period), and different introduction years (from 2000 to 2010). Consistently, we obtained always better fits when the initial year was before 2005 (see Fig SD2), and prevalence levels in 2000 were rather low (1% to 5%). Importantly, our approach allows comparing the likelihood of different introduction years from 2000 on. Until approximately 2005, this likelihood is roughly the same, and then continuously drops down until the present (see Fig D2).

In sum, these two hypotheses, (1) sigmoidal *vs* constant-fraction, and (2) exponential expansion, combined provided empirically-based trajectories for, first, the number of sexual workers (SW), and second, the prevalence of the disease within the total SW population. These two series of empirically generated data, along with accurate adult population time series data per city, were then compared with model-generated trajectories to search for disease model parameters able to produce comparable results. Here we only show results for the *sigmoidal-exponential* hypothesis. Fig SD1 (and shows a good agreement between model-generated trajectories (green shading), and the yearly data (in circles), generated according to the plausible assumptions underlying this hypothesis. Average parameter values for these ensemble trajectories are given in Tables SB1 and SB2.

E. Projected Trajectories

In order to project trajectories, the numerical integration of the full system requires annual time-dependent parameters, namely future recruitment and mortality rates (F_X, F_Y, δ_X, and δ_Y) from 2016 to 2033. These values were extrapolated from the same demographic life tables under the assumption that mortality and fertility rates maintain observed trends between 2000 and 2016 (see Fig SE1).

Because we did not directly use raw data to compare with model output, but generated first time series of pseudo-data compatible with the observations at hand, it is very important to clarify in which terms, and under which conditions, the calculation of projected trajectories was conducted. Table SE1 summarizes the hypotheses under which each ensemble of parametric configurations was obtained. In Figs. SE2 and SE3, we show the model projections up to year 2033 calculated (panels b) for a number of parameter combinations that provide a good fit to data for the period 2000-2016 (see panels a). Similar projections are obtained for other cities (Fig. SE6). Each parameter combination produces a single deterministic trajectory. The ensemble of trajectories is used to calculate percentile values. Upper and lower broken lines represent the 90% and 10% percentile values, respectively. The thicker line in the middle represents 50% percentile values. Data for the period between 2000 and 2016 are represented by circles. They have been generated under the sigmoidal hypothesis (H_5, see Table SD1) to be compatible with the demographic expansion in Madagascar in this period. True data are highlighted with error bars (orange) representing confidence intervals (see years 2014 and 2017 in middle panels in both figures).

We also explored the impact of circumcision practices in Madagascar since it is known that this practice, strongly embedded in Madagascar culture, reduces the probability for males of acquiring the infection from infectious females. This was done by constraining parameter searches within the possible parameter range for p_{YX}, which controls this infection probability once a sexual contact has occurred. "Low p_{YX}" means that searches were conducted in the range $[0, 4\,10^{-4}]$, while "high p_{YX}" involve that searches were conducted in the range $[0, 10^{-3}]$ for this particular parameter (see Table SE1 and Fig SE4). When we compare results, under the same overall hypothesis (H_5), for "high p_{YX}" *vs* "low p_{YX}", we observed that the effect of circumcision on the transmission probability from infectious women to healthy men (p_{YX}) is not very important. For the rest of model parameters, parametric configurations were searched within boundaries, as indicated in Table SA1. Model projections

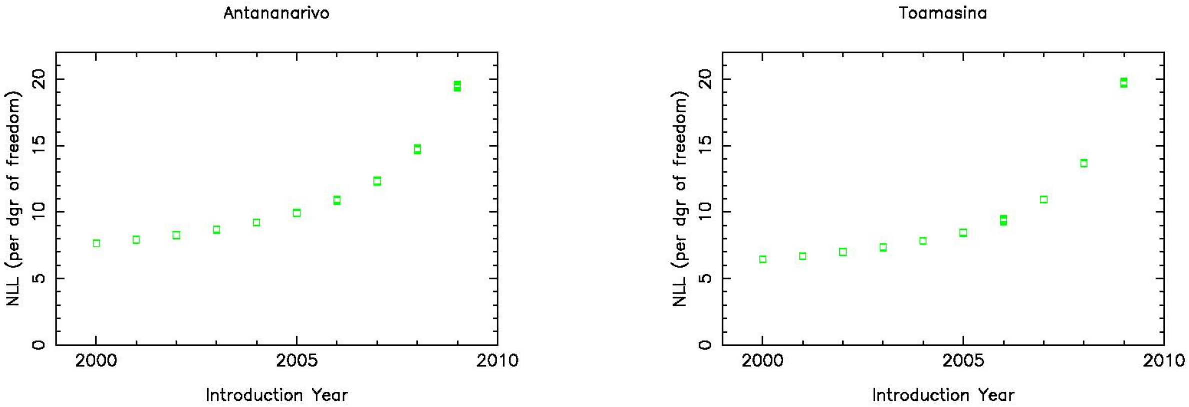

Fig. SD2. Negative Loglikelihood values per degree of freedom. According to this, the estimation procedure is better if the introduction year happens to be before 2005. We found consistent results for the other cities as well.

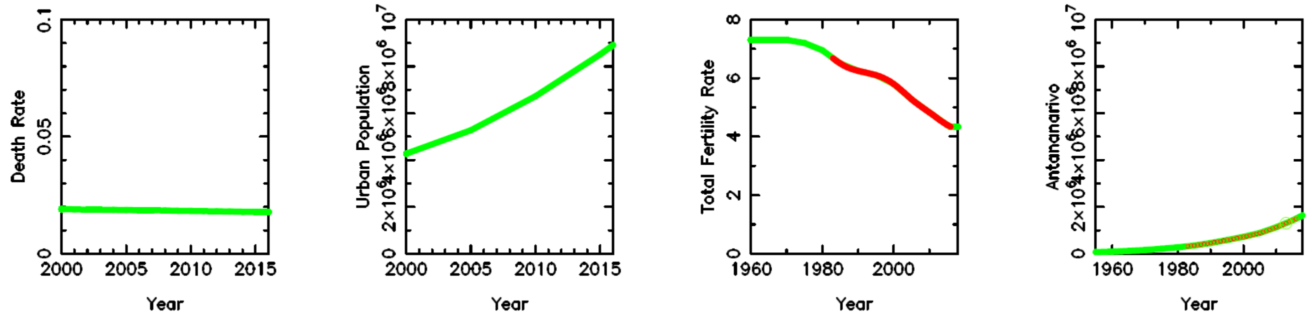

(a) Observed trends

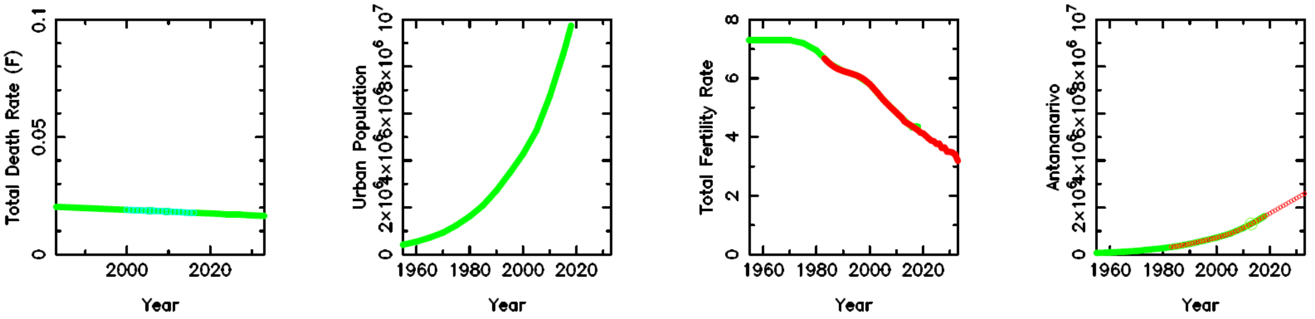

(b) Extrapolation

Fig. SE1. Death rates, population, and fecundity extrapolated into the future up to 2033 based on trends between 2000 and 2016. The upper panel shows 2000-2016 trends and the lower panel extrapolated values. The same procedure was applied to extrapolate age-dependent mortality from life tables between 2000 and 2016 required to extrapolate recruitment rates, according to Eq (SC3).

Table SE1. A summary of the explored hypotheses under which pseudo-data were generated and parameter searches (between 2000 and 2016) were then conducted. In grey, we show the combination that produced the greatest consistency levels between model predictions, data at hand, and the hypotheses.

		Constant Fraction		Sigmoidal	
		$p_{Y X}$		$p_{Y X}$	
		low	high	low	high
$f = W/X$	≈ 2014				
	≈ 2017				

obtained from parametric configurations under the two situations resulted very similar. Here, results for Toamasina (SE4) and Antananarivo (SE5) are shown. A slight effect can be seen in disease prevalence in men. Other cities showed a similar pattern.

Although different hypothesis led to similar conclusions, the best likelihoods were obtained under the sigmoidal hypothesis, for high p_{YX}, and fraction of sex workers ($f = W/X$) similar to those in 2017 (combination represented by the shaded cell in Table SE1). It means that this combination of hypotheses maximized the consistency between available data at hand and model specification. Results in the main text correspond precisely to these hypotheses (Sigmoidal H_5-H_6, see Table SD1).

Parameter searches were conducted by minimizing the negative loglikelihood (see Eq (SD1)). Up to 10^8 different parameter combinations were sampled at random within ranges given by Table SA1. Each initial random combination was then used to seed a simplex algorithm (21) constrained within the prescribed parameter ranges (see Table SA1), which led to different optimal parametric configurations. The ensemble of optimal configurations obtained in this way was further filtered to keep only those that were only 2 points apart from the best one in their negative loglikelihood values.

Some parameter distributions within this filtered ensemble of optimal configurations were quite constrained by the information at hand, while other were less. Optimal distributions appeared to be similar across the different hypothesis and led to the same qualitative conclusions. In Fig SE7, we compare box plots across a number of hypotheses for the whole set of model parameters. The first subplot corresponds to a dummy parameter multiplying recruitment rates F_X and F_Y, which was added as a consistency check. If recruitment rates were correctly estimated to match the temporal evolution of the adult population in every city, as was explained in previous sections A.2 and B, then the optimization procedure for the full disease transmission model, which includes true parameters plus this dummy factor, should only retain parameter configurations where such a factor is equal to 1, as it was the case.

F. Data Sources and Code

Here we show compiled disease and population data (see Tab. SF1) for populations sizes, total sexual worker population, and prevalence of the disease within this group in the main cities of Madagascar after the Institut National de la Statistique, Antananarivo. Other data were compiled from several public sources (16–18, 22). C Code to perform pseudo-data generation under the different hypotheses, run model numerical integrations, conduct parameter estimation from demographic tables, and all theses simplex-based (23), parallel random parameter searches is publicly available in this github site (1).

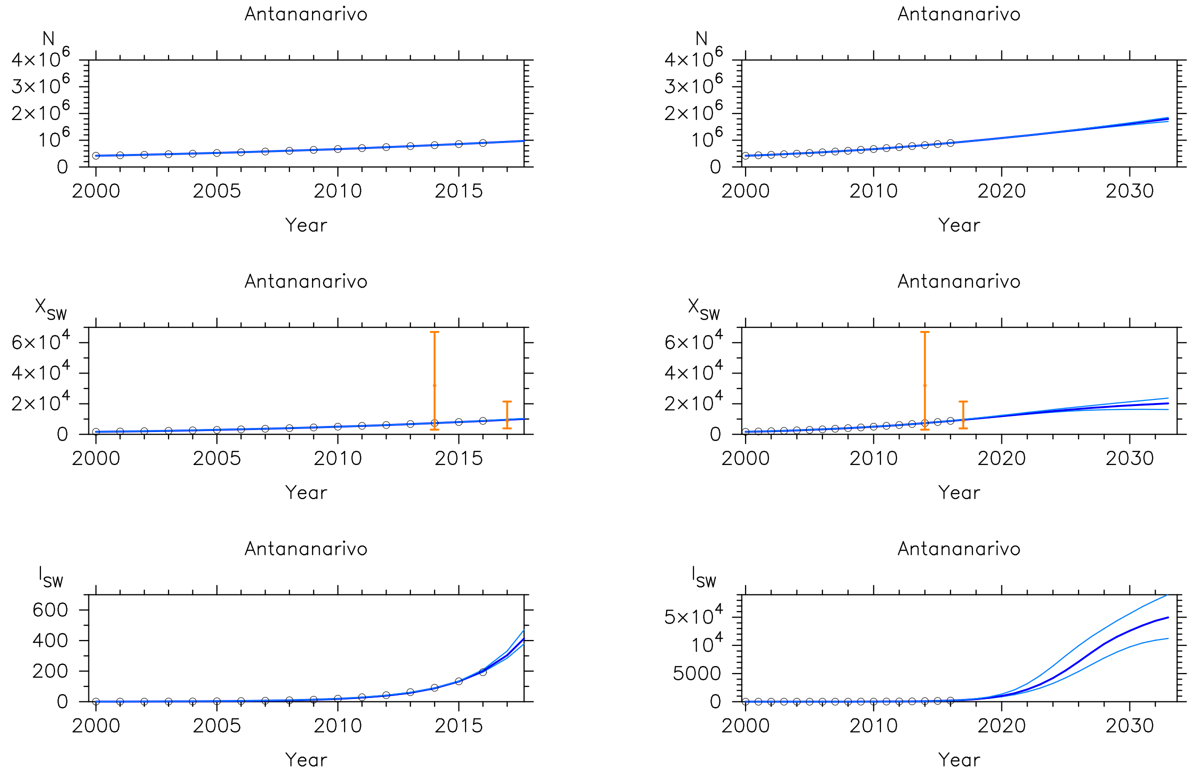

(a) Model fit

(b) Future projection

Fig. SE2. Model fit and projected trends in Antananarivo. N, adult population; X_{SW}, female sexual worker population; I_{SW}, infected individuals within the sexual worker population.

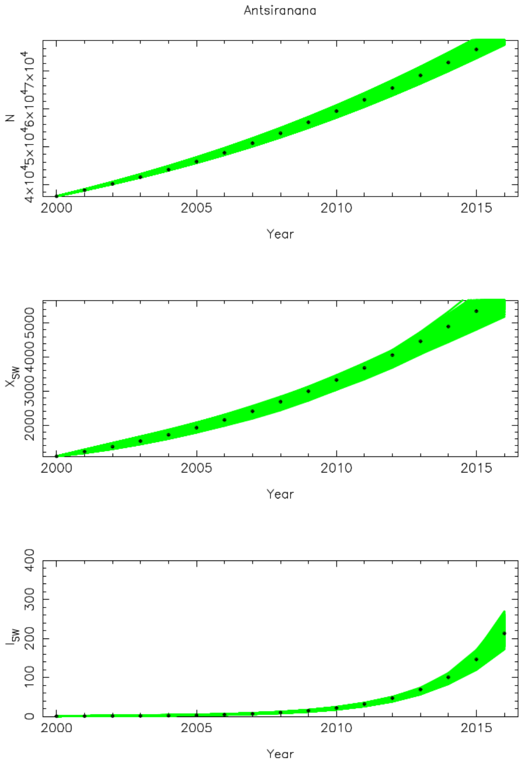

(a) Model fit

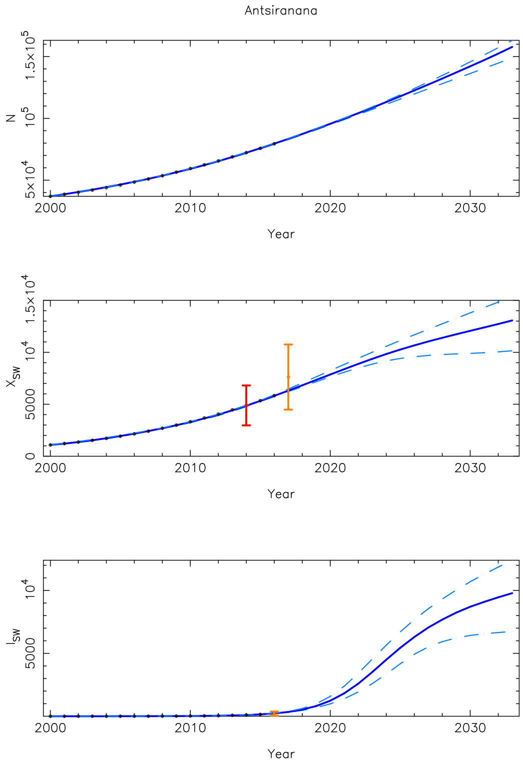

(b) Future projection

Fig. SE3. Model fit and projected trends in Antsiranana. Each parameter combination produces a single deterministic trajectory, which has been represented in green in (a). N, adult population; X_{SW}, female sex worker population; I_{SW}, infected individuals within the sexual worker population.

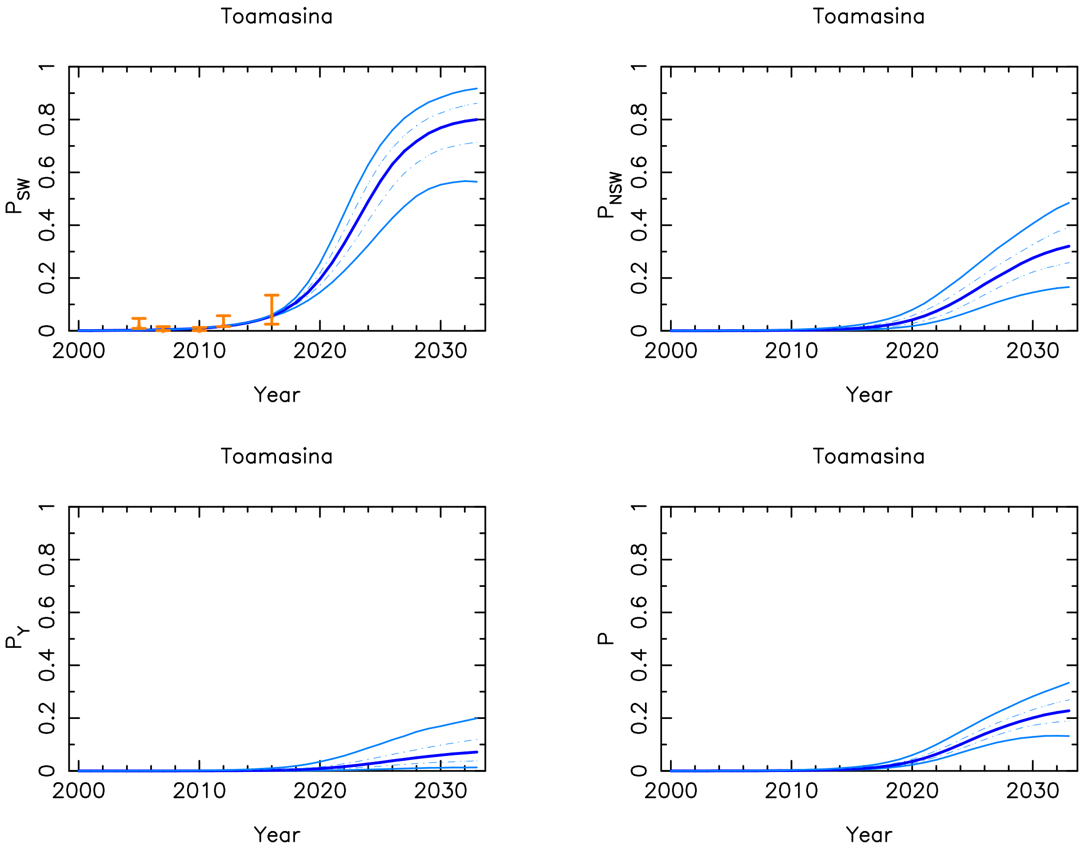

(a) High p_{YX}

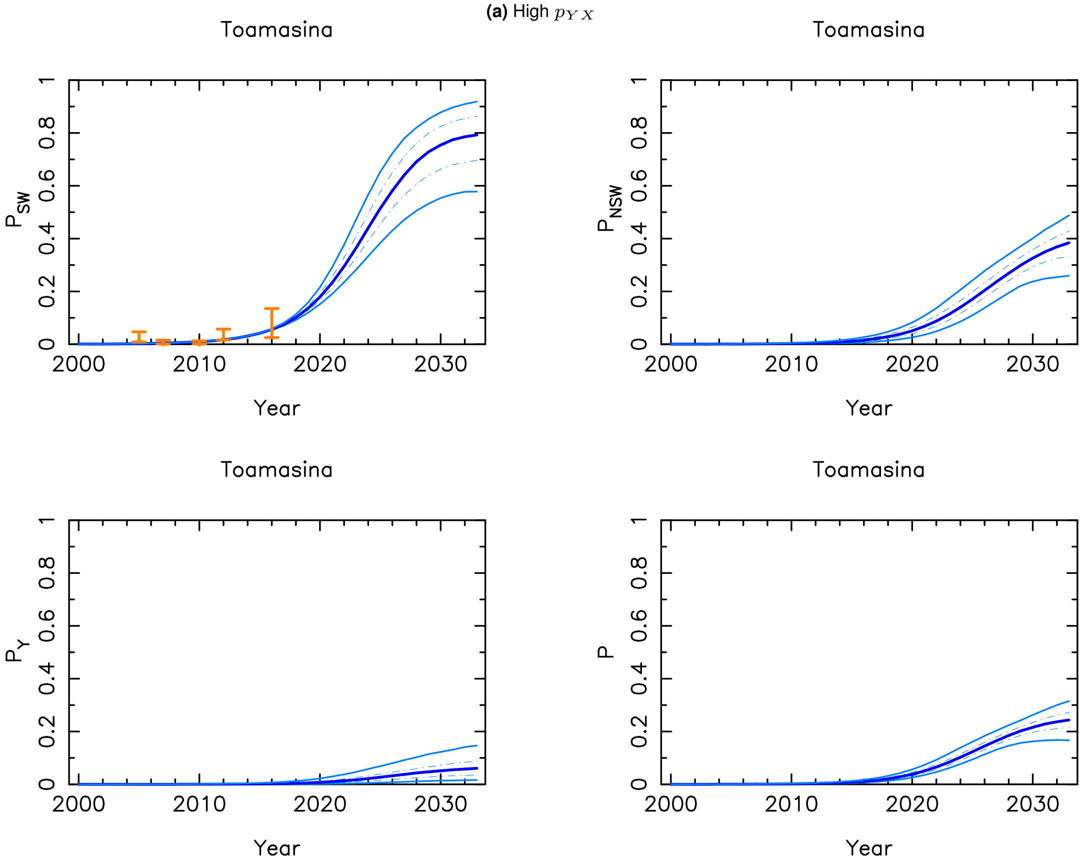

(b) Low p_{YX}

Fig. SE4. The effect of circumcision on the transmission probability from infectious women to healthy men (p_{YX}) in Toamasina.

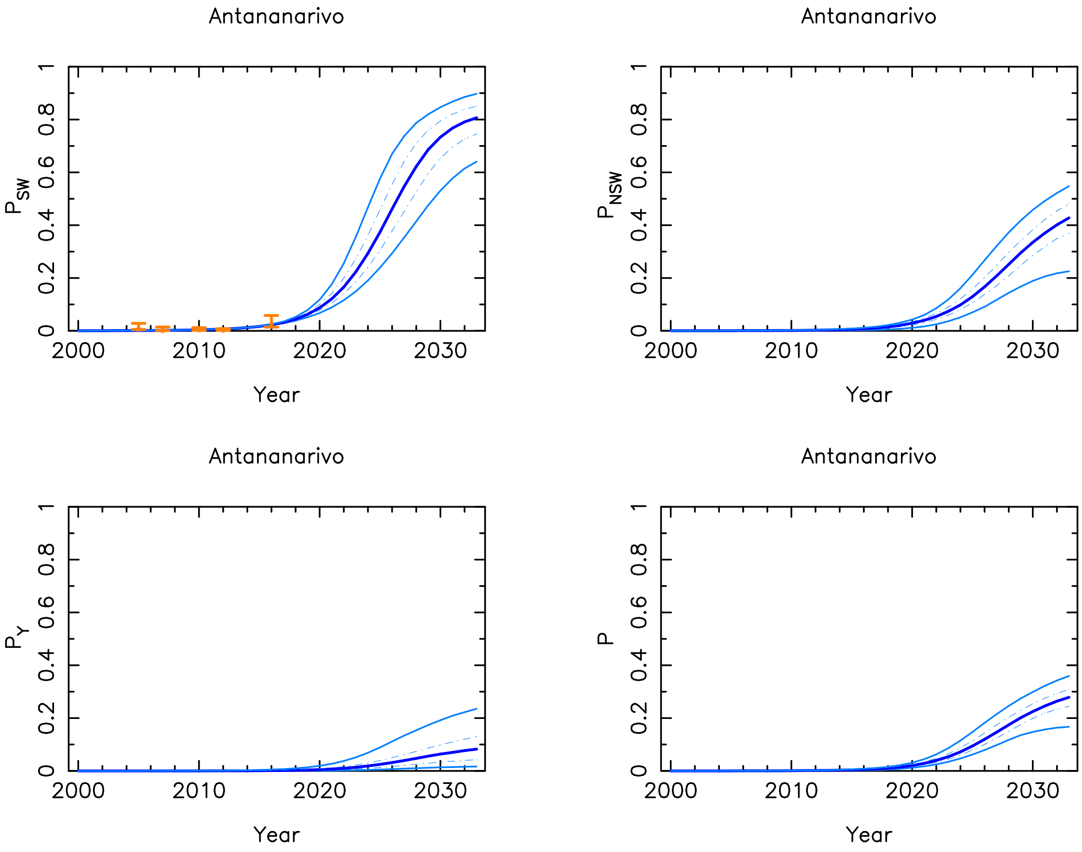

(a) High p_{YX}

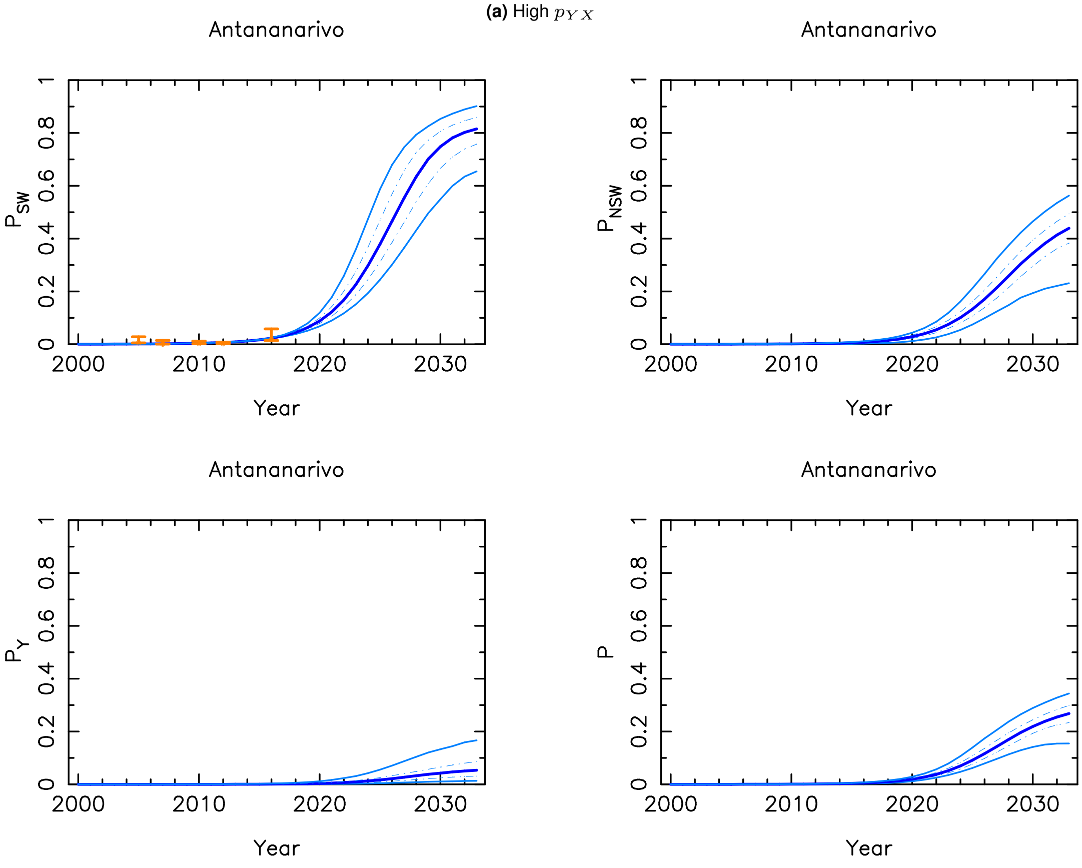

(b) Low p_{YX}

Fig. SE5. The effect of circumcision on the transmission probability from infectious women to healthy men (p_{YX}) in Antananarivo

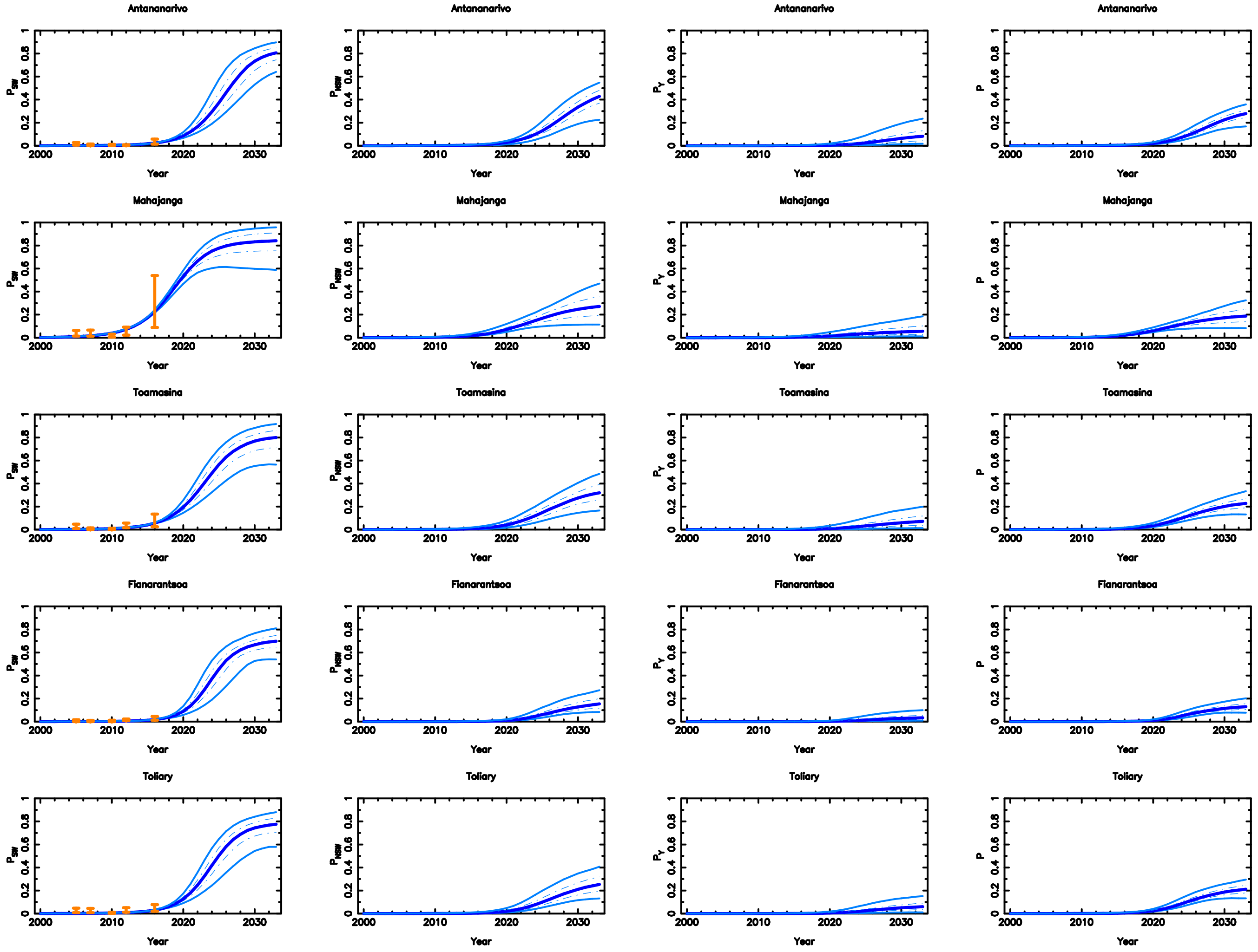

Fig. SE6. Model predicted prevalence across 5 cities in Madagascar for the SW (P_{SW}), non-SW female (P_X), male (P_Y), and overall adult (P) populations are represented in columns 1 to 4, respectively. In addition, true observed data for the prevalence within the SW population are highlighted with error bars representing confidence intervals for years 2005, 2007, 2010, 2012, and 2016 (see first column panels, and Table SF2). The five lines represent 5%, 25%, 50%, 75%, and 95% percentiles from the lowest to the highest values, respectively.

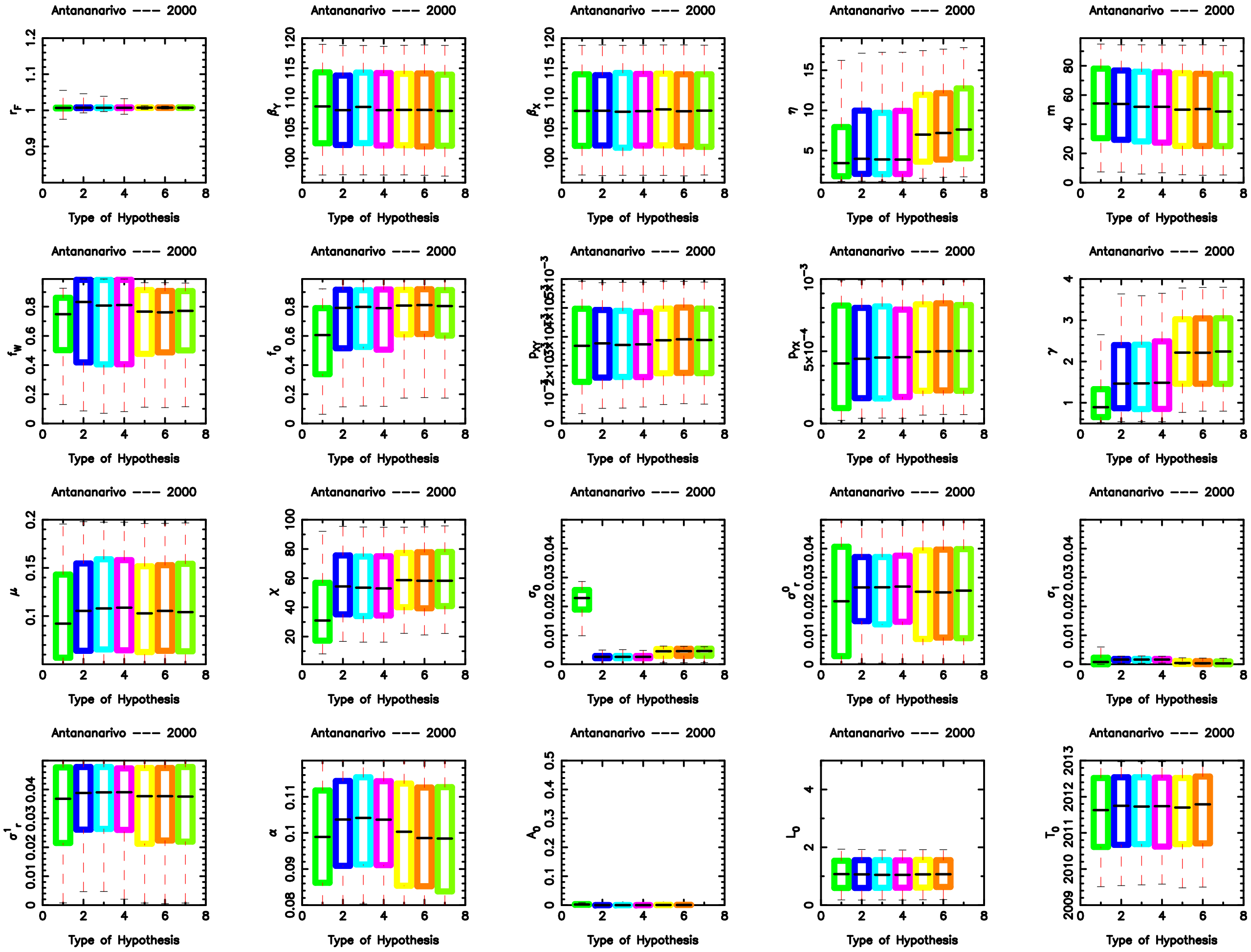

Fig. SE7. Box plots for the different parameter distributions are shown across 7 different sigmoidal hypothesis for the city of Antananarivo. The sigmoidal hypothesis required the prescription of a smoothness parameters L_0 and a threshold year T_0 (see Table SD1). Under this prescription, a time series was generated from 2000 to 2016 compatible with the observed sex worker levels in either 2014 or 2017. Hypothesis compatible were coded as follows $H_n = (L_0^{(n)}, T_0^{(n)})$ at position $x = n + 1$ on the x axis. This is, from left to right, $H_0 = (0.01, 2010)$ at $x = 1$, $H_1 = (0.01, 2010)$ at $x = 2$, $H_2 = (0.01, 2011)$ at $x = 3$, $H_3 = (0.01, 2012)$ at $x = 4$, $H_4 = (0.1, 2010)$ at $x = 5$, $H_5 = (0.1, 2011)$ at $x = 6$, $H_6 = (0.1, 2012)$ at $x = 7$. The one that appears labelled as H_0 corresponds to sigmoidal parameters (0.1, 2011), the same as H_5, but with a fraction of sex workers compatible with that in 2014.

Table SF1. Source data for the total sexual worker population and prevalence in the main cities of Madagascar after the Institut National de la Statistique, Antananarivo.

	Population	Sexual Worker Population				HIV Prevalence					
	2013	2014		2017		2012			2016		
		Min	Max	Min	Max	%	CI		%	CI	
Antananarivo	1300000	28925	35021	4908	12647	0.2	0.0	0.6	1.8	0.4	4
Antsiranana	115015	2978	6812	4489	10749	0.0	0.0	0.0	2.9	0.8	6.5
Mahajanga	220629	2290	5637	571	1516	3.0	0.8	6.2	22.7	13.9	31.2
Toamasina	274667	4289	12336	3638	15120	1.8	0.2	3.9	5.4	2.9	8.1
Fianarantsoa	190318	3415	7795	743	2961	0.9	0.0	1.4	1.2	0.0	3.3
Toliara	156710	4190	11367	1956	7530	1.2	0.0	3.9	2.7	0.7	5.1
Taolagnaro	46000	1392	5059	830	2419	0.0	0.0	0.0	4.1	0.7	9.6
Moramanga	282600	1648	2807	460	1388	0.3	0.0	0.5	0.6	0.0	5.7
Antsirabe	238478	3677	9328	2150	6164	0.0	0.0	0.0	0.0	0.0	0.0
Morondava	123739	1648	3672	502	1733	6.5	3.2	10.0	7.0	3.2	11.3
Nosy Be	73010	7268	14830	2029	4048	–	–	–	9.5	4.5	15.3

Table SF2. Source data for the prevalence within the SW populatiton in the main cities of Madagascar after the Institut National de la Statistique, Antananarivo for the years 2005, 2007, 2010, 2012, and 2016. The number of surveyed cities has increased over this period. In part, this reflects public health concerns about the potential expansion of HIV in the country.

	HIV Prevalence														
	2005			2007			2010			2012			2016		
	%	CI		%	CI		%	CI		%	CI		%	CI	
Antananarivo	0.6	0.1	2.2	0.05	0.0	1.4	0.3	0.1	0.9	0.2	0.0	0.6	1.8	0.4	4
Antsiranana	2.8	1.2	5.3	0.05	0.0	1.6	0.3	0.1	0.9	0.0	0.0	0.0	2.9	0.8	6.5
Mahajanga	2.0	0.7	4.3	2.1	0.9	4.5	0.7	0.2	2.4	3.0	0.8	6.2	22.7	13.9	31.2
Toamasina	1.3	0.4	3.4	0.05	0.0	1.5	0.05	0.0	1.2	1.8	0.2	3.9	5.4	2.9	8.1
Fianarantsoa	0.05	0.0	1.6	0.01	0.0	1.1	0.02	0.0	1.2	0.9	0.0	1.4	1.2	0.0	3.3
Toliara	1.2	0.3	3.5	1.2	0.4	3.3	0.3	0.1	0.9	1.2	0.0	3.9	2.7	0.7	5.1
Taolagnaro	–	–	–	0.3	0.01	1.9	0.3	0.1	0.9	0.0	0.0	0.0	4.1	0.7	9.6
Moramanga	–	–	–	–	–	–	–	–	–	0.3	0.0	0.5	0.6	0.0	5.7
Antsirabe	–	–	–	–	–	–	–	–	–	0.0	0.0	0.0	0.0	0.0	0.0
Morondava	–	–	–	–	–	–	–	–	–	6.5	3.2	10.0	7.0	3.2	11.3
Nosy Be	–	–	–	–	–	–	–	–	–	–	–	–	9.5	4.5	15.3

References

1. D Alonso, The SICA model for HIV-AIDS transmission in Madagascar (https://github.com/vankampen92/PROJECT_HIV-AIDS_MADAGASCAR) (2020).
2. RM Anderson, RM May, *Infectious Diseases of Humans. Dynamics and Control.* (Oxford University Press, Oxford), (1991).
3. T Hollingsworth, R Anderson, C Fraser, HIV-1 Transmission, by Stage of Infection. *The J. Infect. Dis.* **198**, 687–693 (2008).
4. H McCallum, N Barlow, J Hone, How should pathogen transmission be modelled? *Trends Ecol. Evol.* **16**, 295–300 (2001).
5. EW Fiebig, et al., Dynamics of HIV viremia and antibody seroconversion in plasma donors: Implications for diagnosis and staging of primary HIV infection. *Aids* **17**, 1871–1879 (2003).
6. P Bacchetti, AR Moss, Incubation period of AIDS in San Francisco. *Nature* **338**, 251–253 (1989).
7. WHO, World Health Organization. global health observatory data repository (http://apps.who.int/gho/data/node.home) (2018) Accessed: 2018.02.01.
8. MJ Wawer, et al., Rates of HIV-1 transmission per coital act, by stage of HIV-1 infection, in Rakai, Uganda. *J. Infect. Dis.* **191**, 1403–1409 (2005).
9. P Patel, et al., Estimating per-act HIV transmission risk: a systematic review. *AIDS* **28**, 1509–1519 (2014).
10. RH Gray, et al., Probability of HIV-1 transmission per coital act in monogamous, heterosexual, HIV-1-discordant couples in Rakai, Uganda. *Lancet* **357**, 1149–1153 (2001).
11. SD Pinkerton, Probability of HIV transmission during acute infection in Rakai, Uganda. *AIDS Behav.* **12**, 677–684 (2008).
12. D Alonso, A Dobson, M Pascual, Critical transitions in malaria transmission models are consistently generated by superinfection. *Philos. Transactions Royal Soc. B: Biol. Sci.* **374**, 20180275 (2019).
13. EO Omondi, RW Mbogo, LS Luboobi, Mathematical analysis of sex-structured population model of HIV infection in Kenya. *Lett. Biomath.* **5**, 174–194 (2018).
14. O Diekmann, JAP Heesterbeek, MG Roberts, The construction of next-generation matrices for compartmental epidemic models. *The J. Royal Soc. Interface* **7**, 873–885 (2010).
15. RA Alsallaq, et al., Quantitative assessment of the role of male circumcision in HIV epidemiology at the population level. *Epidemics* **1**, 139–152 (2009).
16. CIA, Central Intelligence Agency (https://www.cia.gov/library/publications/the-world-factbook/fields/2018.html) (2018) Accessed: 2019.01.01.
17. WHO, World Halth Organization (http://apps.who.int/gho/data/node.home) (2018) Accessed: 2019.01.01.
18. Instat, Institute de Statistique de Madagascar (https://www.instat.mg) (2018) Accessed: 2019.01.01.
19. MA Randretsa, Evolution and structure of the population of Madagascar. *Demogr. Afr.* **48–49**, 51–60 (1985).
20. M Raberahona, et al., Is Madagascar at the edge of a generalised HIV epidemic? Situational analysis. *Sex. Transm. Infect.* **97**, 27–32 (2021).
21. B Gough, *GNU Scientific Library Reference Manual - Third Edition.* (Network Theory Ltd.), 3rd edition, (2009).
22. Worldometers, Worldometers (https://www.worldometers.info/world-population/madagascar-population/) (2018) Accessed: 2019.01.01.
23. JA Nelder, R Mead, A simplex method for function minimization. *Comput. J.* **7**, 308–313 (1965).
